# Black glucose-releasing silicon elastomer rings for fed-batch operation allow measurement of the oxygen transfer rate from the top and optical signals from the bottom for each well of a microtiter plate

**DOI:** 10.1186/s12896-023-00775-9

**Published:** 2023-03-02

**Authors:** Sarah Sparviero, Laura Barth, Timm Keil, Carl Dinter, Christoph Berg, Clemens Lattermann, Jochen Büchs

**Affiliations:** 1 Aachener Verfahrenstechnik – Biochemical Engineering, RWTH Aachen University, Forckenbeckstr. 51, 52074 Aachen, Germany; 2 Kuhner Shaker GmbH, Kaiserstraße 100, 52134 Herzogenrath, Germany

**Keywords:** BioLector, µRAMOS, Online monitoring, Fed-batch, High-throughput, Screening, *E. coli*, *H. polymorpha*, Polymer-based nutrient feeding, FeedPlate

## Abstract

**Background:**

In industrial microbial biotechnology, fed-batch processes are frequently used to avoid undesirable biological phenomena, such as substrate inhibition or overflow metabolism. For targeted process development, fed-batch options for small scale and high throughput are needed. One commercially available fed-batch fermentation system is the FeedPlate^®^, a microtiter plate (MTP) with a polymer-based controlled release system. Despite standardisation and easy incorporation into existing MTP handling systems, FeedPlates^®^ cannot be used with online monitoring systems that measure optically through the transparent bottom of the plate. One such system that is broadly used in biotechnological laboratories, is the commercial BioLector. To allow for BioLector measurements, while applying the polymer-based feeding technology, positioning of polymer rings instead of polymer disks at the bottom of the well has been proposed. This strategy has a drawback: measurement requires an adjustment of the software settings of the BioLector device. This adjustment modifies the measuring position relative to the wells, so that the light path is no longer blocked by the polymer ring, but, traverses through the inner hole of the ring. This study aimed at overcoming that obstacle and allowing for measurement of fed-batch cultivations using a commercial BioLector without adjustment of the relative measurement position within each well.

**Results:**

Different polymer ring heights, colours and positions in the wells were investigated for their influence on maximum oxygen transfer capacity, mixing time and scattered light measurement. Several configurations of black polymer rings were identified that allow measurement in an unmodified, commercial BioLector, comparable to wells without rings. Fed-batch experiments with black polymer rings with two model organisms, *E. coli* and *H. polymorpha*, were conducted. The identified ring configurations allowed for successful cultivations, measuring the oxygen transfer rate and dissolved oxygen tension, pH, scattered light and fluorescence. Using the obtained online data, glucose release rates of 0.36 to 0.44 mg/h could be determined. They are comparable to formerly published data of the polymer matrix.

**Conclusion:**

The final ring configurations allow for measurements of microbial fed-batch cultivations using a commercial BioLector without requiring adjustments of the instrumental measurement setup. Different ring configurations achieve similar glucose release rates. Measurements from above and below the plate are possible and comparable to measurements of wells without polymer rings. This technology enables the generation of a comprehensive process understanding and target-oriented process development for industrial fed-batch processes.

**Supplementary Information:**

The online version contains supplementary material available at 10.1186/s12896-023-00775-9.

## Background

Biotechnological products are already part of our everyday life, and with ongoing research, the respective product range increases even further. Process development for industrial biotechnological fermentations is a necessary step towards identifying suitable strains as well as optimal cultivation conditions for the production of biotechnological products. For this, a large number of variables have to be tested, and the current standard method is still based on trial-and-error [1]. Applying high-throughput systems (HTPS) in small scale is almost mandatory for a time- and cost-effective screening.

Microtiter plates (MTP) are commonly used for screening on a small scale. The most common ones used for microbial cultivations allow up to 96 parallel cultivations [2–5]. Hence, throughput increases and the required time reduces, while resources are minimised, as only micro to millilitres of medium per well are required. Automated handling systems for microtiter plates are available, such as the RoboLector^®^ (Beckman Coulter GmbH, Krefeld, Germany). These systems allow processes to be further standardised and throughput to be increased even more [6, 7].

Next to high-throughput, online monitoring presents another important challenge in process development. It enables a detailed observation and in-depth understanding of a process. The acquired knowledge can be used to design more stable and robust processes [8]. In addition, online monitoring can decrease workload, as sampling during cultivation may no longer be necessary [9, 10]. There are commercially available solutions for online monitoring in microtiter plates, such as the BioLector (Beckman Coulter GmbH, Krefeld, Germany). In addition, newly developed prototypes, such as the µRAMOS or µTOM, expand the range of measurable parameters [11, 12]. The employed methods include, among others, measurements of dissolved oxygen tension (DOT), back-scattered light, pH, fluorescence and oxygen transfer rates (OTR) [11, 13, 14].

Microtiter plates are state of the art in the first stages of process development and are routinely used for batch fermentations [3]. However, many industrial processes are carried out using fed-batch operating conditions [15, 16]. Applying fed-batch can avoid a lot of adverse biological or physical phenomena, such as substrate or osmotic inhibition, catabolite repression or overflow metabolism [17–21]. Yet, technical and economically feasible methods for fed-batch operation are often lacking in process development. Accordingly, batch is still customary practice in the earlier stages of process development, although fed-batch is used on production scale. This results in a major gap between industrial scale and process development. Strains can behave very differently regarding product formation, when cultivated in batch or fed-batch mode [22]. In the worst case, a screening in batch mode would lead to selected strains that perform poorly in fed-batch mode [23, 24]. To close this gap, increasing efforts have been made in recent years to realise fed-batch operation mode on small scale. One approach involves completely novel cultivation vessels, such as described by Puskeiler et al. (2005) or Bower et al. (2006) [25–27]. One commercially available system is the Ambr^®^ 15 (Sartorius AG, Göttingen, Germany). However, its main drawback lies in the high investment cost, as the system cannot be integrated into other existing microtiter plates handling systems [28]. So far, only DOT and pH online measurement data are available [29].

A BioLector-based fed-batch system was described by Funke et al. (2010) and is now commercially available under the name of BioLector XT (Beckman Coulter GmbH, Krefeld, Germany) [30, 31]. Feeding is realised with specially designed disposable microtiter plates and microfluidic pumps. Online measurement of DOT, back-scattered light, pH and fluorescence during cultivation is possible, allowing for a broader range of parameters to be measured, compared to the before-mentioned Ambr^®^ 15 system. The microtiter plates with the microfluidic pumps are exclusively designed for applications in the BioLector XT, so investment costs can be high.

More flexible fed-batch systems are based on enzymatic release. This approach is not restricted to specific cultivation vessels. The commercially available EnBase system (Enpresso^®^ GmbH, Berlin, Germany) was first described by Panula-Perälä et al. (2008) and facilitates feeding through a controlled glucose release by enzymatic degradation of starch [32–34]. A major disadvantage is the dependency of the enzymatic glucose release on cultivation conditions, e.g., cultivation temperature and pH [35].

Other release systems employ polymer matrices instead of enzymes. First described for shake-flasks in 2006 by Jeude et al., Scheible et al. (2010) introduced a polymer matrix into the microtiter plate, located at the bottom of the well [23, 36]. Keil et al. (2019) further investigated and characterized the use of this technology [37]. Fed-batch microtiter plates called FeedPlates^®^ are commercially available (Kuhner Shaker, Herzogenrath, Germany). However, such microtiter plates cannot be used with online-measuring systems that measure across the bottom of the plate, such as the BioLector, because the polymer matrix optically blocks the bottom of the well. Hence, in this case, only online monitoring through the top of the well, such as by the µRAMOS or the µTOM technologies, can be employed [28]. Accordingly, the set of parameters available is limited to OTR (and CTR). To overcome this obstacle, Habicher et al. (2019) proposed using polymer rings at the bottom of the plate with a sufficiently large inner diameter, allowing measurements using the BioLector [38]. However, this is only possible, if the measuring position of the optical fibre under the bottom of the microtiter plate is adjusted accordingly by the user, increasing the effort before each cultivation.

This study proposes an improved version of fed-batch microtiter plates with polymer rings that allow for measurement by the BioLector and the µRAMOS without any adjustments, making this method fast and easy to use.

## Materials and methods

### Strains

In this work, one bacterial model strain of *E. coli*, was used. *E. coli* BL21 DE3 pRhotHi-2-EcFbFP produces a Flavin mononucleotide (FMN)-based fluorescent protein (FbFP), which is under the control of the *lac* operon [6, 39]. *H. polymorpha* RB11 pC10-FMD was chosen as a yeast model strain, expressing a GFP protein under the control of the FMD promoter [23, 40]. Both strains have already been intensively investigated and successfully used in small-scale fed-batch processes. It has been shown before that GFP and FbFP at high concentrations may interfere with the pH-measurement [41]. However, this interference only appears at late fermentation times. For the aim of this paper this effect is of no great relevance. Therefore, the two mentioned strains were considered suitable for the demonstration of the polymer rings [23, 37, 38, 42].

### Media

For media preparation, chemicals of analytical grade were applied and purchased from one of the following manufacturers: Carl Roth GmbH & Co. KG (Karlsruhe, Germany), Sigma-Aldrich Chemie GmbH (Steinheim, Germany), Merck KGaA (Darmstadt, Germany).

All cultivations of *E. coli* in this work were performed in modified Wilms-MOPS medium. The mineral medium was originally introduced by Wilms et al. (2001) for fed-batch cultivations with *E. coli* BL21 [43]. The used modified version has already been shown in several papers to be a suitable mineral medium for batch and fed-batch cultivations of *E. coli* [9, 12, 42, 44]. The base solution consists of 6.98 g/L (NH_4_)_2_SO_4_, 3 g/L K_2_HPO_4_, 2 g/L Na_2_SO_4_ and 41.85 g/L 3–(N-morpholino) propane sulfonic acid (MOPS). The pH value was adjusted with 10 mM NaOH solution to 7.5. All solutions were sterilised before microbial cultivation at 121 °C for 20 min. Shortly before cultivation, 10 mL/L of a MgSO_4_ (50 g/L MgSO_4_ · 7 H_2_O) solution and 1 mL/L each of a sterile filtered thiamine solution (10 g/L thiamine hydrochloride) and a sterile filtered trace element solution were added to the base solution. The MgSO_4_ solution was sterilised at 121 °C for 20 min. The trace element solution consists of 0.54 g/L ZnSO_2_ · 7 H_2_O, 0.48 g/L CuSO_4_ · 5 H_2_O, 0.30 g/L MnSO_4_ · H_2_O, 0.54 g/L CoCl_2_ · 6 H_2_O, 41.76 g/L FeCl_3_ · 6 H_2_O, 1.98 g/L CaCl_2_ · 2 H_2_O, 33.40 g/L Na_2_EDTA · 2 H_2_O (Titriplex III). For batch cultivations, the medium was complemented with 20 g/L glucose. Fed-batch cultivations were conducted without initial glucose.

*H. polymorpha* was cultivated in Syn6-MES medium. This mineral medium was developed for fed-batch cultivations with *H. polymorpha* and has successfully been used in several fed-batch cultivations [37, 45–47]. The base solution consists of 7.66 g/L (NH_4_)_2_SO_4_, 1 g/L KH_2_PO_4_, 27.3 g/L 2-(N-morpholino) ethane sulfonic acid (MES), 3 g/L MgSO_4_ · 7 H_2_O, 3.3 g/L KCl, 0.33 g/L NaCl. The base solution was adjusted to a pH of 6.0 with 10 mM NaOH. Shortly before cultivation, the following solutions were added to the base solution: 10 mL/L of a calcium chloride solution (100 g/L calcium chloride · 2 H_2_O), and 10 mL each of a microelement solution, a vitamin solution and a trace element solution. The microelement solution consists of 6.65 g/L Na_2_EDTA · 2 H_2_O, 6.65 g/L (NH_4_)_2_Fe(SO_4_)_2_ · 6 H_2_O, 0.55 g/L CuSO_4_ · 5 H_2_O, 2 g/L ZnSO_4_ · 7 H_2_O and 2.65 g/L MnSO_4_ · H_2_O. The vitamin solution consists of 0.04 g/L d-biotin and 13.35 g/L thiamine chloride. D-biotin is dissolved in 10 mL of a 1:1 water-isopropyl alcohol, while thiamine is dissolved in demineralised water. Both solutions were then mixed together. The trace element solution consists of 0.065 g/L NiSO_4_ · 6 H_2_O, 0.065 g/L CoCl_2_ · 6 H_2_O, 0.065 g/L H_3_BO_3_, 0.065 g/L KI and 0.065 g/L Na_2_MoO_4_ · 6 H_2_O. All solutions were sterile filtered except for the calcium chloride solution, which was sterilised at 121 °C for 20 min. Fed-batch cultivations were conducted without any initial glucose.

### Microbial cultivation

All precultures were grown in 250 mL shake flasks in an orbital shaker with a shaking frequency (n) of 300 rpm, a shaking diameter (d_0_) of 50 mm and a temperature (T) of 30 °C.

For *E. coli* cultivations, flasks were filled with 10 mL of Wilms-MOPS medium, inoculated with 50 µL of a cryo culture (initial OD_600nm_ = 0.08) and cultivated for approximately 18 h.

For precultures of *H. polymorpha*, flasks were filled with 10 mL of Syn6-MES medium, inoculated with 200 µL of a cryo stock and grown for approximately 20 h.

All microbial main cultivations were conducted, if not stated otherwise, in triplicates in 48 round well microtiter plates (without optodes: MTP-R48-B, with DOT and pH optodes: MTP-R48-BOH; Beckman Coulter GmbH, formerly m2p-labs, Krefeld, Germany) and shaken by 1000 rpm at a shaking diameter of 3 mm.

For batch cultivations with *E. coli* in microtiter plates, each well was filled with 0.8 mL inoculated Wilms-MOPS medium (initial OD_600nm_ = 0.5) and cultivated at a temperature of 37 °C.

Fed-batch cultivations were performed in microtiter plates with glucose-releasing polymer rings. Cultivations were performed at a temperature of 37 °C for *E. coli* and 30 °C for *H. polymorpha* in wells containing 0.8 mL inoculated medium (initial OD_600nm_ = 0.5).

The OTR measurement was conducted using the µRAMOS-device, invented by Flitsch et al. (2016) [11]. Measurements of DOT, scattered light, pH and fluorescence were conducted using a commercial BioLector (Beckman Coulter GmbH, Krefeld, Germany). The following settings were chosen (λ_ex_: excitation wavelength; λ_em_: emission wavelength): Gain 15, λ_ex, em_ = 620 nm for scattered light, λ_ex_ = 520 nm and λ_em_ = 600 nm for dissolved oxygen tension (DOT), λ_ex_ = 470 nm and λ_em_ = 525 nm for pH, λ_ex_ = 450 nm and λ_em_ = 495 nm for FbFP fluorescence, λ_ex_ = 488 nm and λ_em_ = 520 nm for green fluorescence.

### Polymer rings

**Fig. 1:**
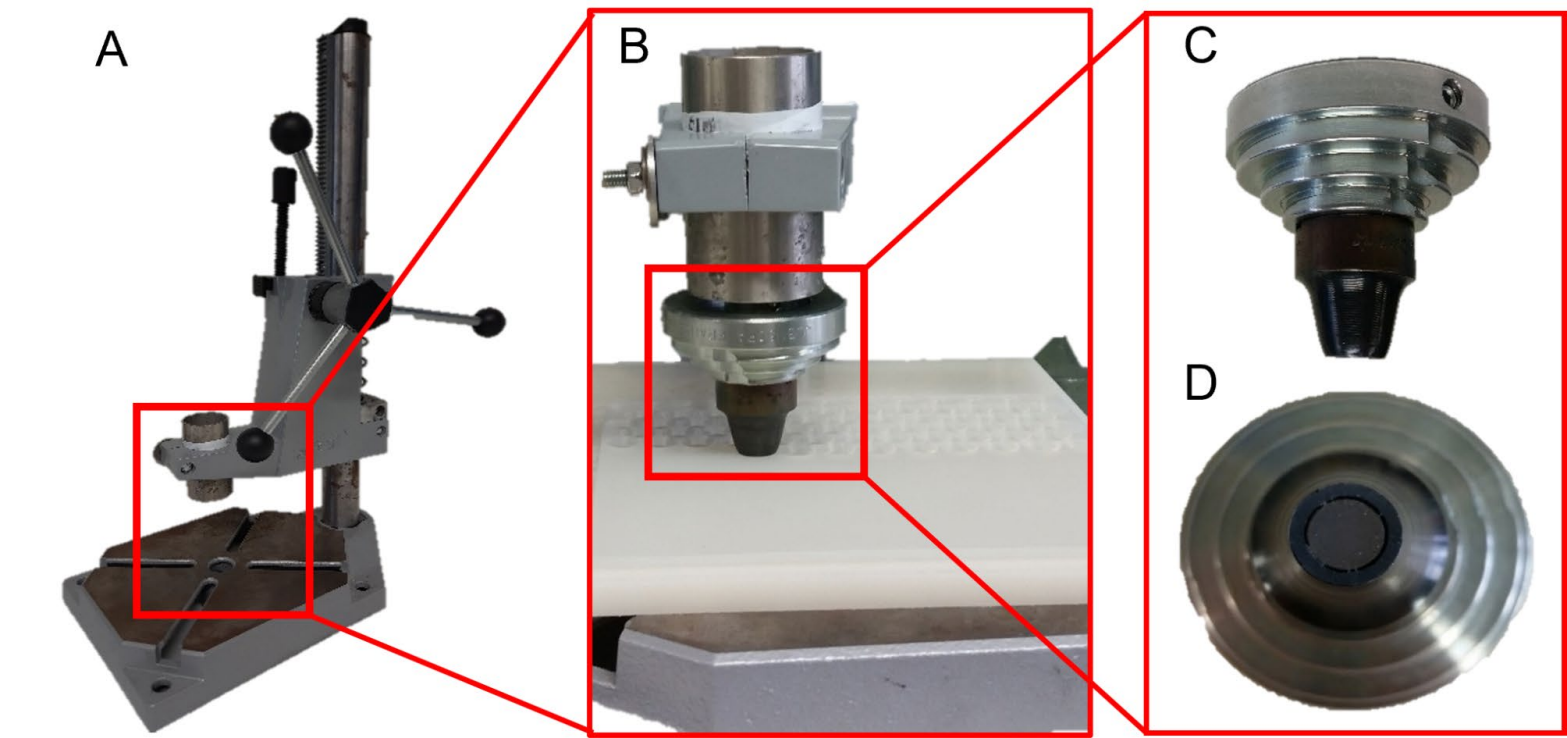
**Pictures of the punching tool applied to fabricate the polymer rings.** (A) Frame with a manual lever. (B) View of the arrangement with frame, punch tool and silicone plate on a plastic plate. (C) Zoom side view of the punch tool. (D) Axial zoom view of the punch tool

Different polymer ring specifications depending on the application were used in this work. For abiotic experiments and biotic batch experiments, polymer rings manually punched from transparent silicone elastomer plates of 3 mm thickness ((C_2_H_6_OSi)_n_, Goodfellow GmbH Germany, art. no. SI303300) were used. Figure 1 pictures the self-constructed punching tool.

**Fig. 2:**
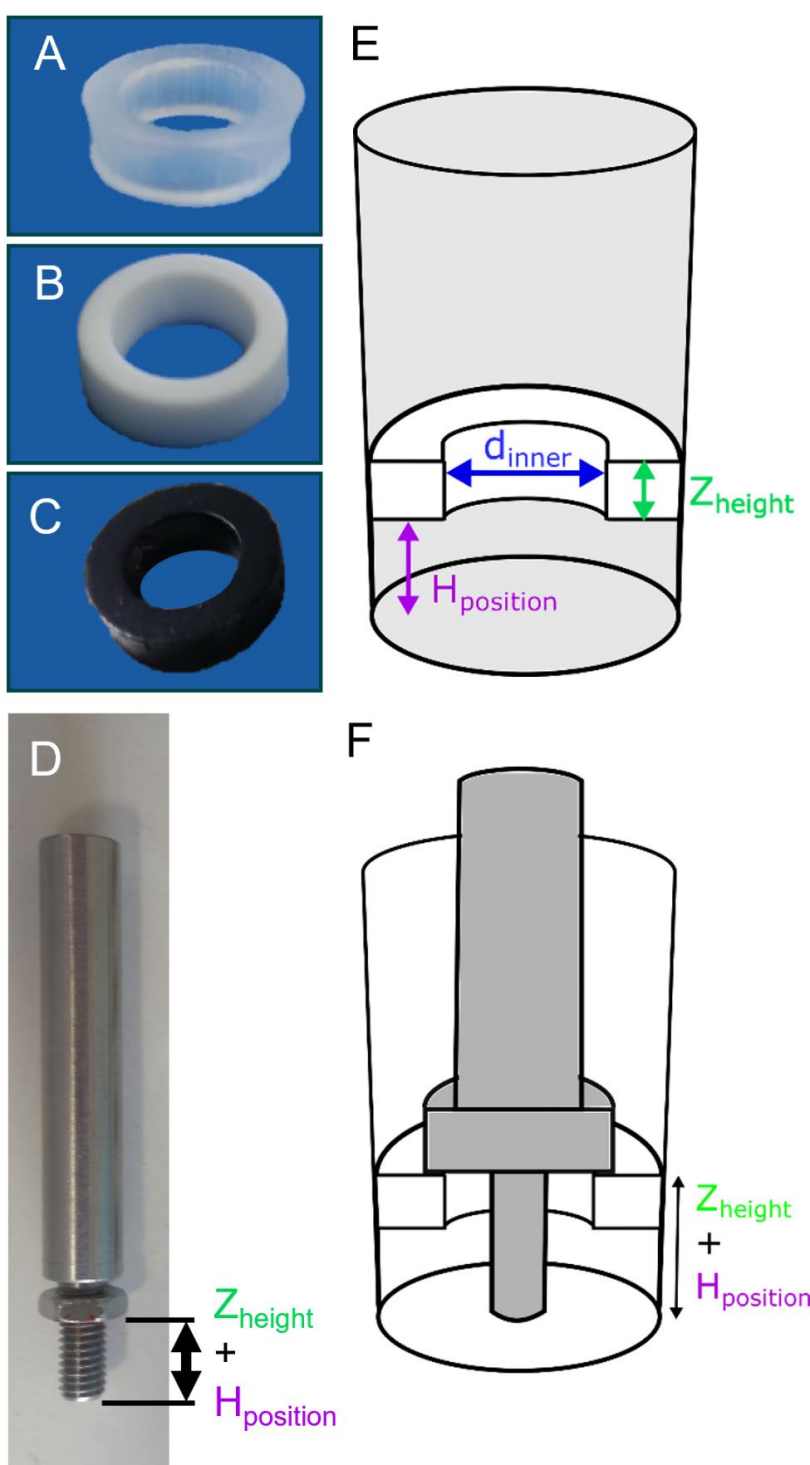
**Feed rings and positioning tool.** (A) Punched transparent ring without glucose crystals. (B) Punched white ring with encapsulated glucose crystals. (C) Punched transparent ring without glucose crystals sprayed with black lacquer. (D) Tool to position the polymer rings in the wells of a microtiter plate. (E) Illustration of the well geometries with a positioned feed ring. Ring configuration is determined by the ring inner diameter (d_inner_), the height of the ring (Z_height_) and the vertical position of the ring in the well (H_position_). (F) Schematic view of how the positioning tool is applied to position a polymer ring in a well of a microtiter plate

To investigate the influence of the polymer ring colour on measurement during abiotic experiments and biotic batch experiments, transparent rings (Fig. 2A) were sprayed with black lacquer (OBI Group Holding, art. no. 3,468,485, Fig. 2C). White polymer rings were manufactured for comparison by punching rings out of a 3 mm silicone plate with 25% glucose (provided by Kuhner Shaker GmbH, Germany, Fig. 2B). All rings are defined by their outer diameter of 12 mm, their variable inner diameter (d_inner_) and height (Z_height_, Fig. 2E). Inner diameters of 7 or 8 mm were chosen, as Habicher et al. (2019) already showed that smaller inner diameters cover parts of the fluorescent optodes for DOT and pH measurement on the bottom of the microtiter plate. Smaller inner diameters lead to a higher annulus. Larger inner diameters lead to a smaller annulus and were, hence, unstable and tended to break [38]. Z_height_ was altered by stacking up two or three 3 mm thick rings in a well above another, gaining Z_heights_ of 6 and 9 mm, respectively. The third parameter is the vertical ring position H_position_ of the ring in a well, representing the distance between the ring and the bottom of the well. A self-constructed positioning tool with adjustable 3 mm high nuts enabled precise ring positioning (Fig. 2D). To position the ring, the nuts were stacked according to the ring height (Z_height_) and adjusted according to the desired vertical position (H_position_). The ring is then placed above the well and pushed downwards using the positioning tool to its appropriate vertical position (H_position_, Fig. 2F). Four different H_position_ were investigated: 0 mm, 1.5 mm, 3.5 and 5.5 mm. Higher positions were disregarded, as complete wetting of the polymer rings by the liquid could not be assured. After inserting these polymer rings into microtiter plates, they were used for abiotic experiments and batch experiments.

For fed-batch cultivations, 48-round well microtiter plates (Beckman Coulter GmbH, MTP-R48-B and MTP-R48-BOH, Krefeld, Germany) were customised by Kuhner, with 3 mm high polymer rings made of a cross-linked siloxane elastomer with embedded glucose crystals that has been described before [37]. These rings were coloured with black dye, which is known to be non-toxic. The plates equipped with polymer rings were then sterilised by β-radiation and used for fed-batch cultivations.

### Determination of the maximum oxygen transfer capacity

To determine the maximum oxygen transfer capacity (OTR_max_) for different polymer ring configurations, *E. coli* batch cultivations were conducted using the µRAMOS-device [11]. The chosen amount of glucose of 20 g/L leads to an oxygen limitation, indicated by a plateau in the oxygen transfer rate [48]. The OTR_max_ is defined as the maximum of the plateau [49]. From triplicates, an OTR_max_ mean value was calculated, and for comparison, wells without polymer rings were evaluated. The distribution of polymer ring configurations over the microtiter plate was randomly generated using Origin 2019 (OriginLab). The principle of this OTR_max_ determination with a biological model system is shown in Figure S1.

### Volume correction of the calculation of the oxygen transfer rate

The calculation of the oxygen transfer rate (OTR) applied in the µRAMOS device depends on the gas volume in the well. During the measurement, the volume of the ring (V_polymer ring_) is not considered in the software. Therefore, post-experimental correction is required, as the addition of a ring to the liquid (V_L_ = 800 µL), decreases the remaining volume of the well, the gas volume. For correcting the raw oxygen transfer rate (OTR_raw_) calculated by the µRAMOS- device, this decrease in the gas volume has to be taken into account, leading to a volume-corrected oxygen transfer rate (OTR_real_). The calculation was done using Eq. (1):

1 $$OT{R_{real}} = OT{R_{raw}} \cdot \frac{{{V_{well}} - {V_{polymer\,ring}} - {V_L}}}{{{V_{well}} - {V_L}}}$$

For the microtiter plate used in this work, the volume per well is 3.7 mL (V_well_). The ring shape was assumed to be an ideal hollow cylinder and its volume (V_polymer ring_) was calculated using Eq. (2), taking the ring height (Z_height_) as well as the inner (d_inner_) and outer ring diameter (d_outer_ = 12 mm) into account:

2 $${V_{polymer\,ring}} = {Z_{height}} \cdot \pi  \cdot \frac{{d_{outer}^2 - d_{inner}^2}}{4}$$

The correction applying Eqs. (1) and (2) results in around 7% lower values of OTR_real_ than OTR_raw_.

### Determination of mixing time

**Fig. 3:**
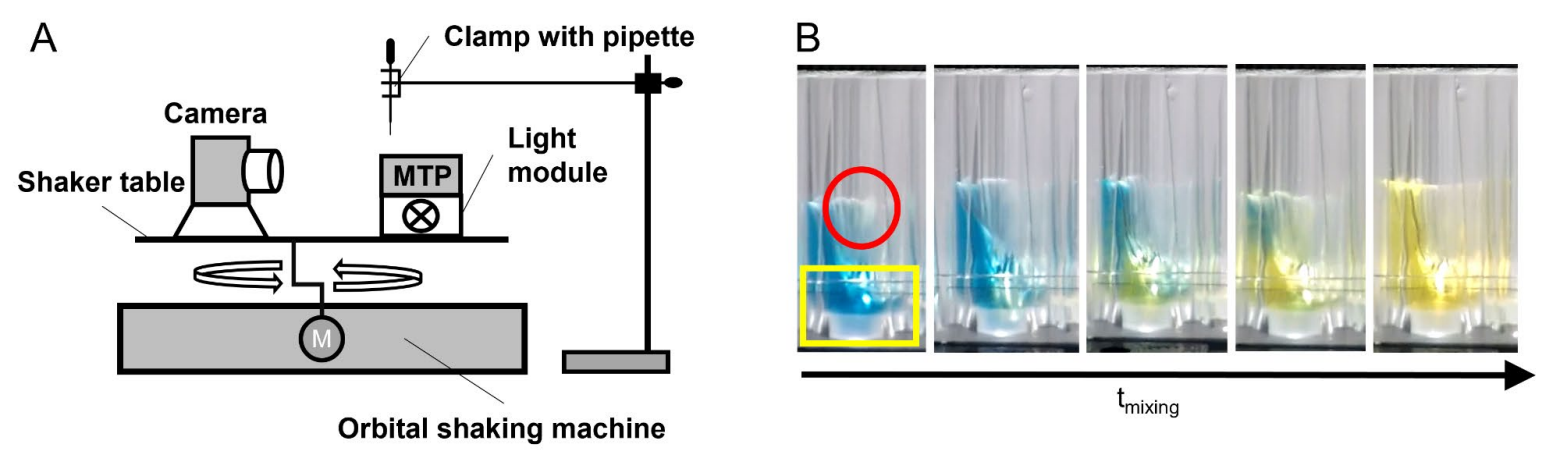
**Schematic experimental setup for mixing time determination and exemplary evolution of a mixing time experiment**. (A) A GoPro Hero 4 camera is mounted onto a shaker table facing a clear microtiter plate (MTP) on a light module. A pipette filled with sulphuric acid is fixed with a clamp above the microtiter plate, which is equipped with polymer rings. The wells of the microtiter plate were filled with a 3 vol-% bromothymol blue solution. By adding 0.2 M sulphuric acid, a colour change from blue to yellow can be observed. (B) The frames taken from a video show the characteristic colour change. For evaluation with the self-written MATLAB^®^-tool, two areas are depicted. The starting point of mixing time (t_mixing_) determination is defined by the time point the acid drop passes the maximum liquid height (shown by red circle). The yellow square depicts the area for evaluation of the colour change. Experimental conditions: T = 37 °C, n = 1000 rpm, d_0_ = 3 mm, V_L_ = 0.8 mL, transparent polymer ring, H_position_ = 0 mm, Z_height_ = 3 mm

To determine the mixing time a colour change method was chosen. The mixing process was recorded via a high-frame-rate camera and a software-assisted assessment [50–52]. The method is based on recommendations from literature, such as by Meusel et al. (2016), Cabaret et al. (2007) and Li et al. 2020 [53–55]. Using a high-frame-rate camera allows for a more precise determination of the mixing time than by the traditional determination by naked eye and a stop watch. The experimental set-up is shown in Fig. 3A. A clear microtiter plate (kindly provided by Beckman Coulter GmbH, Krefeld, Germany) was mounted on a light module on a shaker tray of an orbital shaking machine. The light module is an in-house customized microtiter plate, equipped with 48 Nichia NFSW757GT Horticulture-LEDs [56]. A camera (GoPro Hero 4, GoPro Inc., San Mateo (California), USA) was mounted in front of the microtiter plate on the tray. The chosen colour change method is based on the reaction of bromothymol blue with sulfuric acid. Therefore, a solution of 3 vol-% bromothymol blue (1 g/L bromothymol blue in 20 vol-% ethanol) and demineralised water was adjusted to pH 9 with 0.1 M NaOH. Experimental conditions were a filling volume of 0.8 mL, a shaking frequency of 1000 rpm, a shaking diameter of 3 mm, and a temperature of 37 °C. A pipette was installed above the plate with a clamp, allowing the addition of drops of 0.2 M sulfuric acid to the well in every position during shaking. The addition of sulfuric acid to the bromothymol blue solution leads to pH shift and changes the colour from blue to yellow. The mixing time for each polymer ring configuration was evaluated in triplicates, from which a mean value was calculated. The recorded videos with 240 frames per second (fps) were evaluated using a self-written MATLAB^®^ tool (The MathWorks^®^, Inc., Massachusetts, USA). The exemplary evolution of a mixing time experiment with frames taken from recorded videos can be seen in Fig. 3B.

To determine mixing times, the duration of the colour change from blue to yellow had to be extracted from the recorded videos. Corresponding to the approach of Rodriguez et al. (2013), the recorded 8-bit-RGB video files are split into the respective red, green and blue channels [52]. Only the channel best representing the monitored colour change is further processed. In the present work, the red channel shows the largest change, with a clear sigmoidal shape between the start and end of the reaction.

The change in colour is not simultaneous in the entire liquid, but starts localised at the droplet impact position and propagates through the entire liquid. Usually, badly mixed zones exist, requiring a longer time period to fully change colour. Therefore, the processing of the red channel must be responsive to local changes in red channel values. For this reason, the 90th and 10th percentile of the red channel values were calculated for each frame and fitted to a sigmoidal. An exemplary diagram with the calculated data and the fit can be seen in Figure S2. To minimize the data that needs to be processed, only every 20th frame was used for calculation. For the sigmoidal fit, an open source MATLAB^®^-script was used [57]. The 90th and 10th percentile represent the first changes after droplet impact and the last changes due to badly mixed zones, respectively.

The start of the colour change equates to the beginning of the sigmoidal slope of the 90th percentile. This point was approximated as the first frame for which the 2nd derivative of the sigmoidal fit exceeds the maximal value of the 2nd derivative. The end of the colour change equates to the end of the sigmoidal slope of the 10th percentile. Accordingly, it was approximated as the last frame, for which the 2nd derivative of the sigmoidal fit is still smaller than the minimal value of the 2nd derivative. Following this automated process, the start frame was, whenever possible, manually adjusted to the frame, where the droplet impact could be observed.

The mixing time was then calculated by multiplying the number of frames between the start and the end of the colour change by the frame rate of the GoPro, which is 240 fps.

### Variation of the measuring position of the glass fibre relative to the well

The glass fibre of the BioLector measurement system under the microtiter plate has a default setting, that determines, from which position each well is measured (Supplementary material, Fig. S3). The movement of the glass fibre is based on a coordinate system (Figure S3A) that is stored in the BioLector software. The positioning under each well is optimized for regular microtiter plates and is not a parameter to be changed by the user during regular use. To investigate the influence of polymer rings and their colour on the measurement in a commercial BioLector, the position of the glass fibre was altered from its default position from 0 mm up to 6.5 mm offset (Figure S3B). 0 mm offset corresponds to the default position, which is 4.4 mm from the centre of the well (Fig. S3) [38].

### Calculation of the glucose release rate based on respiratory activity in the fed-batch phase

To calculate the glucose release rate based on the respiratory activity of both model organisms in the fed-batch phase, respective equations were established. Equation (3) shows a stoichiometric equation for growing *E. coli* cultures on glucose with an average cell composition estimated by Grosz et al. (1983) [58].

3 $$\eqalign{&{\varvec{C}}_{6}{\varvec{H}}_{12}{\varvec{O}}_{6}+0.66 \varvec{N}{\varvec{H}}_{3}+3.21 {\varvec{O}}_{2} \cr & \to 2.74 \varvec{C}{\varvec{H}}_{1.77}{\varvec{O}}_{0.49}{\varvec{N}}_{0.24} +3.26 \varvec{C}{\varvec{O}}_{2} + 4.56 {\varvec{H}}_{2}\varvec{O}}$$

Equation (4) shows the stoichiometric equation for growing *H. polymorpha* cultures on glucose. An average cell composition like that of *Pichia pastoris* was assumed [59].

4 $$\eqalign{&{\varvec{C}}_{6}{\varvec{H}}_{12}{\varvec{O}}_{6}+0.35 \varvec{N}{\varvec{H}}_{3}+3.54 {\varvec{O}}_{2} \cr & \to 2.43 \varvec{C}{\varvec{H}}_{1.76}{\varvec{O}}_{0.64}{\varvec{N}}_{0.14} +3.57 \varvec{C}{\varvec{O}}_{2}+4.38 {\varvec{H}}_{2}\varvec{O}}$$

Stoichiometric factors were calculated by balancing the elements of the equation. Biomass yields on glucose of 0.38 g_biomass_/g_glucose_ for *E. coli* and 0.35 g_biomass_/g_glucose_ for *H. polymorpha* were assumed [36, 60]. Product formation for *E. coli* and *H. polymorpha* was not included in these equations, because it was either neglectable, as it was not induced, or it was not determined.

During cultivation, the OTR was online monitored. The integral of the OTR equals the amount of consumed oxygen at a specific point of time and is referred to as accumulated oxygen transfer (AOT, see also Fig. S11). Both parameters are volume dependent [11].

Resulting from the balanced stoichiometric Eqs. (3) and (4), the stoichiometric factor for oxygen is for growing *E. coli* cultures 3.21 and for growing *H. polymorpha* cultures 3.54. This number is the molecular amount of oxygen (O_2_) that is needed to convert one mole of glucose according to Eqs. (3) and (4). To calculate the absolute amount of consumed glucose from the absolute amount of consumed oxygen, the accumulated oxygen transfer (AOT, [mmol/L]) is multiplied by the filling volume of the well (V_L_, [L]) and divided by the stoichiometric factor of oxygen:

5 $$\begin{gathered} Glucose\,consumption =  \hfill \\\frac{{AOT \cdot {V_L}}}{{stoichiometric\,factor\,of\,oxygen\,[ - ]}}\,[mmol] \hfill \\ \end{gathered} $$

## Results and discussion

### Determining the influence of different polymer ring configurations on maximum oxygen transfer capacity

Adding polymer rings of various geometries and configurations to wells has two main consequences: (1) altering the liquid height because of fluid displacement and (2) influencing liquid distribution during shaking. Hence, depending on the configuration, polymer rings may influence the formation of the rotating bulk liquid and decrease or increase the resulting oxygen transfer area between air and medium [61–63]. To identify, whether and how the addition of polymer rings to a well changes the oxygen transfer, the maximum oxygen transfer capacity (OTR_max_) for *E. coli* cultivations was determined.

**Fig. 4:**
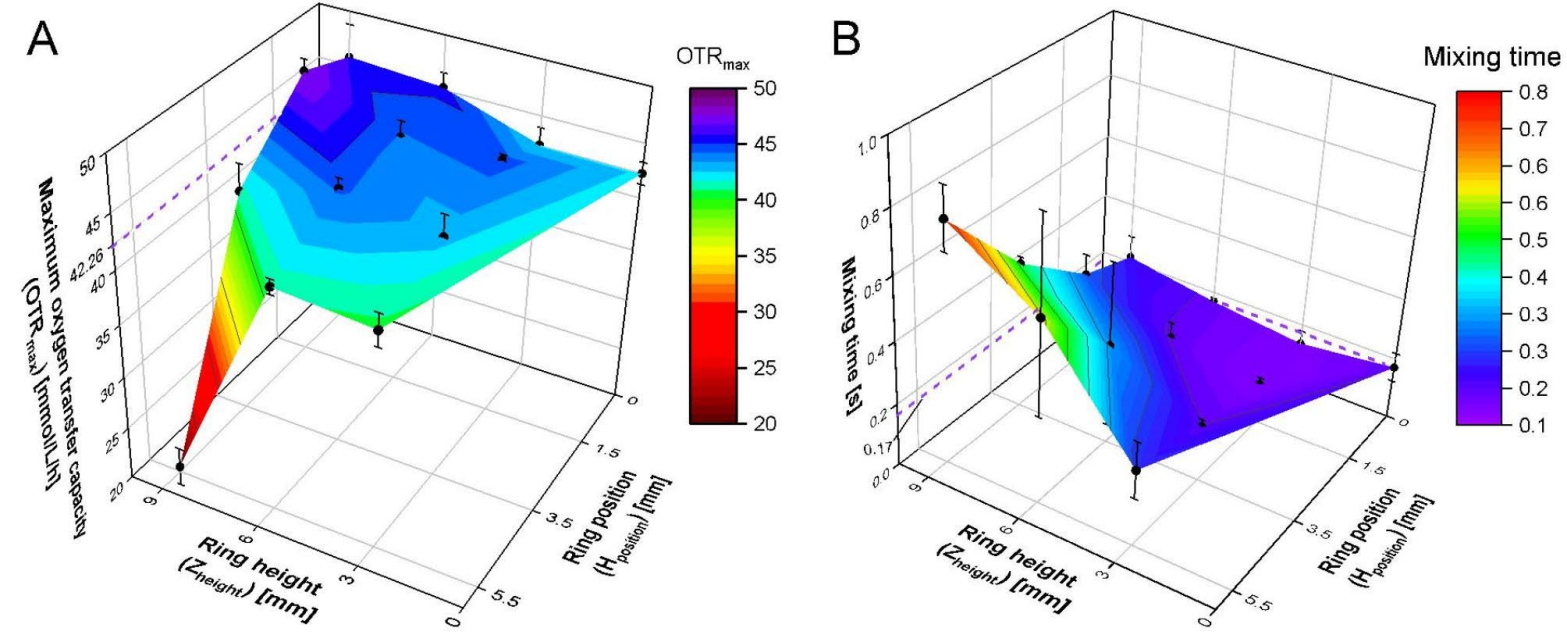
**Influence of polymer rings on the maximum oxygen transfer capacity (OTR**_**max**_**) and the mixing time.** 48 round well microtiter plate, transparent polymer rings without glucose crystals with d_inner_ = 8 mm (Fig. 2A), H_position_ = 0.0 mm, 1.5 mm, 3.5 and 5.5 mm, Z_height_ = 3 mm, 6 and 9 mm. As a control, wells without any polymer rings were evaluated, highlighted in the diagram with a purple dashed line ( **- - -** ). (A) Influence of polymer ring configurations on the OTR_max_. Values were determined for cultivations with *E. coli* in Wilms-MOPS medium with 20 g/L glucose conducted in triplicates. Error bars depict the standard deviation, calculated for each set of triplicates. The oxygen transfer rate (OTR) was determined using the µRAMOS – device. Cultivation conditions: initial pH = 7.5, OD_600 nm, start_ = 0.5, V_L_ = 0.8 mL, T = 37 °C, n = 1000 rpm, d_0_ = 3 mm. (B) Influence of polymer ring configurations on the mixing time. The mixing time was determined in triplicates with a colour change method, illustrated in Fig. 3. Error bars represent the standard deviation, calculated for each set of triplicates. Experimental conditions: V_L_ = 0.8 mL, T = 37 °C, n = 1000 rpm, d_0_ = 3 mm.

Fig. 4A shows the OTR_max_ for polymer rings with d_inner_ = 8 mm. For comparison, the OTR_max_ of 42.26 ± 1.01 mmol/L/h was determined for wells without polymer rings and is represented in the diagram by a dashed violet line. Wells with polymer rings with Z_height_ = 3 or 6 mm have, independent of H_position_, OTR_max_ values comparable to the reference. This, as well, applies for higher polymer rings (Z_height_ = 9 mm) at H_positions_ = 0 and 3.5 mm. Polymer rings of Z_height_ = 9 mm at H_position_ = 1.5 mm show only a minor increase in the OTR_max_ value up to 47.65 ± 1.08 mmol/L/h. The only major deviation from the reference is visible for the configuration Z_heigth_ = 9 mm at H_position_ = 5.5 mm, leading to an OTR_max_ of 20.5 mmol/L/h, only half of the OTR_max_ of the reference.

For those last two ring configurations, the deviation of OTR_max_, compared to the OTR_max_ of the reference, is likely caused by different effects. An increase in OTR_max_ may be caused by a ring acting as a baffle and, therefore, increasing the oxygen transfer area. A decrease in OTR_max_ may be caused by a ring configuration, that reduces the liquid boundary area to the air. The ring acts as a second wall with a smaller inner diameter than the original well. This can lead to a narrower shape of the moving bulk liquid compared to bulk liquids in wells with a bigger inner diameter. Furthermore, the incorporation of rings into the well creates regions within the well with different inner diameters. Therefore, the formation of the typical shape of the bulk liquid during rotation can be hindered. The combination of both phenomena decreases the oxygen transfer area. Comparable results for polymer rings with d_inner_ = 7 mm were obtained (Supplementary Data, Fig. S4A).

To conclude, only one of twelve investigated polymer ring configurations (Z_height_ = 9 mm at H_position_ = 5.5 mm) showed major deviations compared to wells without any polymer rings. Hence, a variety of polymer ring configurations is available for cultivations with comparable OTR_max_ values.

### Determining the influence of different polymer ring configurations on mixing time

As described in the previous section, incorporated polymer rings in wells might act as an obstacle inside the well and, hence, change the liquid distribution during shaking. As this might also influence the mixing time, it was investigated in wells with and without polymer rings by a colour change method. The results for rings with d_inner_ = 8 mm are depicted in Fig. 4B.

The mixing time of wells without polymer rings equals 0.17 s and is highlighted with a dashed violet line. The mixing time increases with increasing Z_height_ as well as H_position_. Wells with polymer rings at H_position_ of 0 and 1.5 mm as well as of Z_height_ = 3 mm at H_position_ = 3.5 mm show only slight variations in comparison to the reference. The highest difference to the reference was detected for wells with Z_height_ = 9 mm and H_position_ = 5.5 mm.

Similar trends for the mixing time using rings with d_inner_ = 7 mm were obtained (Supplementary Data, Fig. S4B). As shown in both diagrams of Fig. 4B and S4B, the standard deviation for longer mixing times is higher than for shorter mixing times. The corresponding ring configurations include higher ring positions (H_position_). By evaluating the videos of the mixing process in slow motion, it is notable that certain ring configurations can hinder the homogenization process by hampering the mixing of certain areas with the rest of the bulk liquid. How fast those areas mix, is also dependent on how the acid droplet enters the bulk liquid, leading to higher standard deviations. A layer formation is undesired for microbial cultivations, as inhomogeneities in the culture broth may lead to unwanted effects and unreproducible results [64]. Therefore, high ring positions with a higher standard deviation should be avoided. Nonetheless, still nine polymer ring configurations (d_inner_ = 7 and 8 mm) have short mixing times of around or under 0.5 s with low standard deviations, comparable to the reference. This makes them suitable for microbial cultivations, where fast mixing and reproducibility are mandatory.

### Investigating the influence of polymer ring colour and configuration on scattered light measurements in a commercial BioLector

**Fig. 5:**
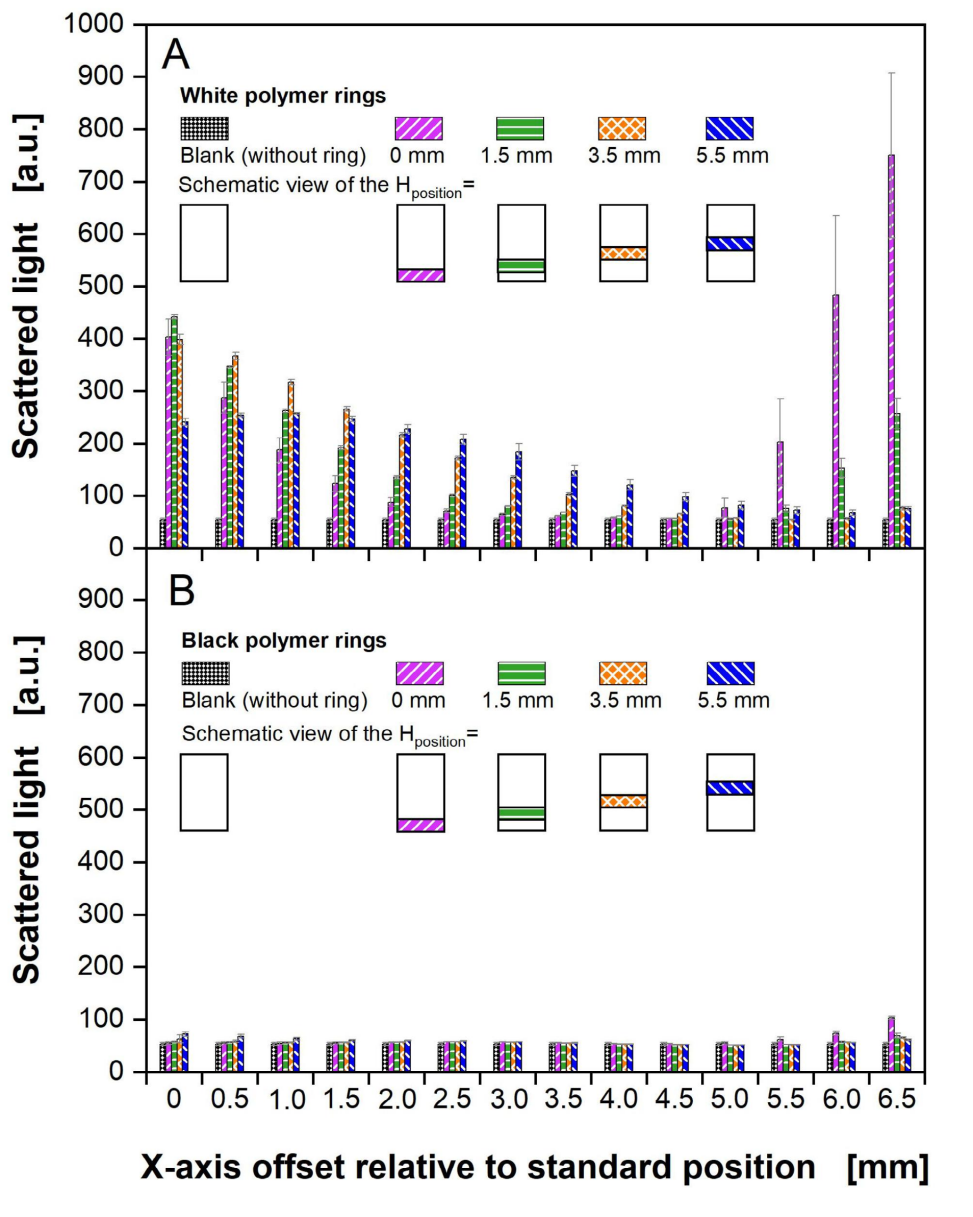
**Influence of differently coloured polymer rings on scattered light measured in a commercial BioLector device.** Polymer rings: (A) white polymer rings with glucose crystals (Fig. 2B) and (B) rings without glucose crystals sprayed with black lacquer (Fig. 2C), both with d_inner_ = 8 mm, Z_height_ = 3 mm, H_position_ = 0.0 mm, 1.5 mm, 3.5 and 5.5 mm. As a control, wells without polymer rings were evaluated. Experimental parameters: V_L_ = 0 mL, T = 37 °C, n = 1000 rpm, d_0_ = 3vmm. Commercial BioLector settings: Gain 15, λ_ex, em_ = 620 nm, three replicates measured three times, position of the X-Y-positioning device (glass fibre) was varied on the X-axis (Fig. S3) with a positive offset of 0 to 6.5 mm relative to the standard position.

Online monitoring of cultivations in microtiter plates is often performed by measurement through the transparent bottom of the plate, such as with the commercially available BioLector [4]. Habicher et al. (2019) already demonstrated that incorporating polymer rings in the wells leads to severe differences in scattered light measurement, caused by the increased back scattering of the white surface of the rings [38]. To overcome this obstacle, they proposed to change the measurement position of the glass fibre under the microtiter plate (Fig. S3B). This adjustment towards a more suitable relative position decreases the back scattering to a level comparable to the reference, but is normally not intended to be changed by the user. Therefore, in this study, it was investigated, whether a change in ring colour would decrease the light scattering and allow for adjustment-free cultivation. Figure 5 shows scattered light measurements in a commercial BioLector with microtiter plates without any liquid filling. Only ring configurations with Z_height_ = 3 mm were investigated, as they have shown the best results for the OTR_max_ and mixing time, compared to wells without polymer rings. For white polymer rings (Fig. 5A), the scattered light is high for the standard measuring position (offset = 0 mm). Increasing the offset by steps of 0.5 mm, the scattered light decreases and is the lowest for an offset position of 4.5 to 5.0 mm. Further increase of the offset position leads again to an increase in scattered light. Those findings are in accordance with the investigations by Habicher et al. (2019) [38].

Figure 5B shows the findings for measurements with polymer rings sprayed with black lacquer. The scattered light for these measurements is similar for all measuring positions with and without polymer rings. Similar results could be obtained using polymer rings with an inner diameter of 7 mm (Fig. S5). Therefore, a black colourisation of polymer rings is suitable for measurement of optically accessible parameters at all investigated positions of the X-Y-positioning device. This ring colourisation allows for an adjustment-free measurement in a commercial BioLector.

### Measuring scattered light of biological batch cultivations with polymer rings

**Fig. 6:**
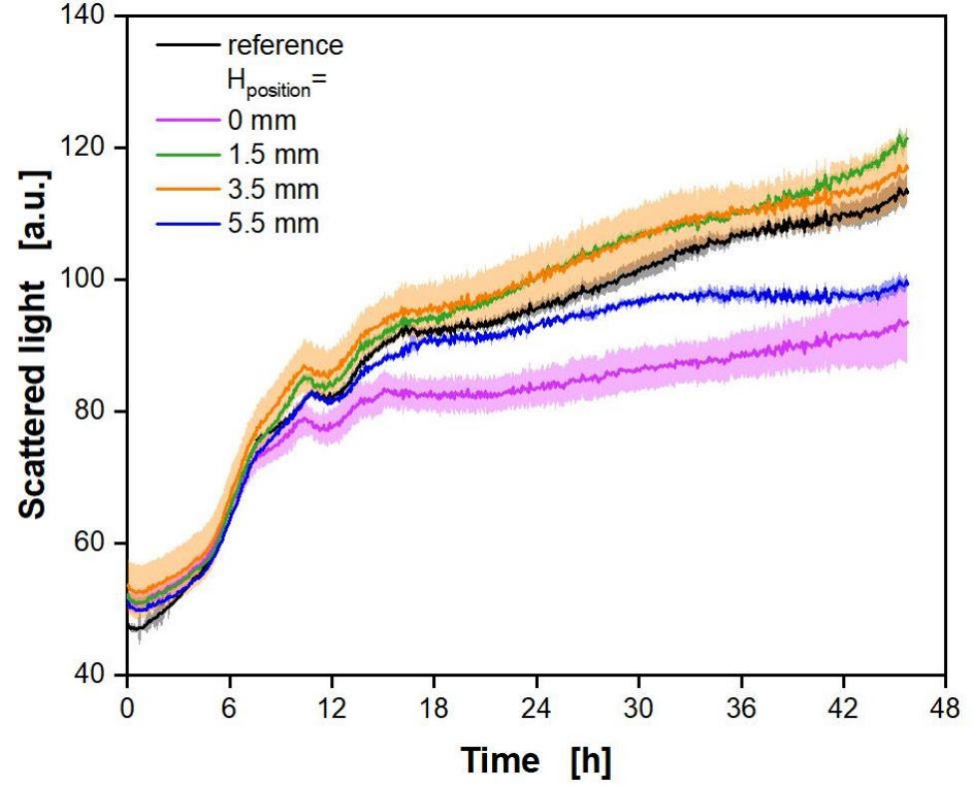
**Influence of the H**_**position**_**of black polymer rings on scattered light measurement during cultivation.** Cultivation was performed with *E. coli* in Wilms-MOPS-medium with 20 g/L glucose using a 48-well microtiter plate (MTP-R48-B) in triplicates (n = 3). Polymer rings: ring without glucose crystals sprayed with black lacquer (Fig. 2C), d_inner_ = 8 mm, Z_height_ = 3 mm, H_position_ = 0.0 mm, 1.5 mm, 3.5 and 5.5 mm. As a control, wells without polymer rings were evaluated. Experimental parameters: initial pH = 7.5, V_L_ = 0.8 mL, T = 37 °C, n = 1000 rpm, d_0_ = 3 mm, OD_600 nm, start_ = 0.5. Commercial BioLector settings: Gain 15, λ_ex,em_ = 620 nm, glass fibre in standard position (offset = 0 mm, Figure S3)

The previous chapter showed the suitability of black polymer rings for adjustment-free measurements in a commercial BioLector. In a next step, the scattered light measurement of biotic cultivations with *E. coli* with polymer rings, sprayed with black lacquer and without glucose crystals, was investigated. As biomass was the only target parameter, no product formation was actively induced. The results are presented in Fig. 6. As a reference, wells without a polymer ring were evaluated. The same polymer ring configurations as in Fig. 5 were applied.

The cultivations align perfectly between cultivation hours 3 and 7. Within the first three hours, the curves differ only slightly. The cultivations with H_position_ = 1.5 and 3.5 mm evolve similar to the reference cultivation. The cultivations with a polymer ring at H_position_ = 0 or 5.5 mm start to differ from the reference after 7 to 12 h cultivation time, respectively. The further increase in scattered light is lower than for the reference, leading to final values that are up to 18 to 12% lower, respectively. Nonetheless, the characteristic features of the curve are comparable to the reference, such as the exponential growth in the beginning and the kink at about 12 h. A toxic effect of the black lacquer on the bacteria, which might be responsible for the deviations, can be excluded. The exponential growth phase is very similar for all cultivations, with or without a ring. At the same time, some cultivations with polymer rings show a comparable course of the scattered light to the reference. In the case of a toxic effect caused by the lacquer, all cultivations should be equally affected and should deviate from the reference to a similar extent. This is not the case here and, hence, a toxic effect can be excluded. With these cultivation conditions and initial glucose concentration, the metabolic activity should decline after roughly 20 h. At this time point, glucose and produced overflow metabolites, such as acetate, usually have been consumed. Therefore, further growth, and, thus, an increase in the scattered light signal is not expected [65]. Further increase in scattered light after cells reached the stationary phase has already been observed before, and can be explained by changes in morphology [66, 67]. This applies equally to all cultivations. Similar results were obtained with rings with an inner diameter of 7 mm (Supplementary Data, Fig. S6).

With this experiment, the suitability of black polymer rings in batch cultivations in combination with measurements of scattered light in a commercial BioLector was shown. Polymer rings at certain configurations (H_position_ = 1.5 and 3.5 mm) lead to values comparable to reference cultivations in wells without polymer rings, especially during the metabolically active phases. Two other configurations at H_position_ = 0 and 5.5 mm showed a lower increase in scattered light after the exponential phase. The reason for this is yet unclear, as no specific interference could be detected in abiotic experiments (see Fig. 5). However, all cultivations share the same characteristic features in the evolution of the scattered light signal over time.

### Applying polymer rings for biological fed-batch cultivations with the prokaryotic model organism *E. coli*

**Fig. 7:**
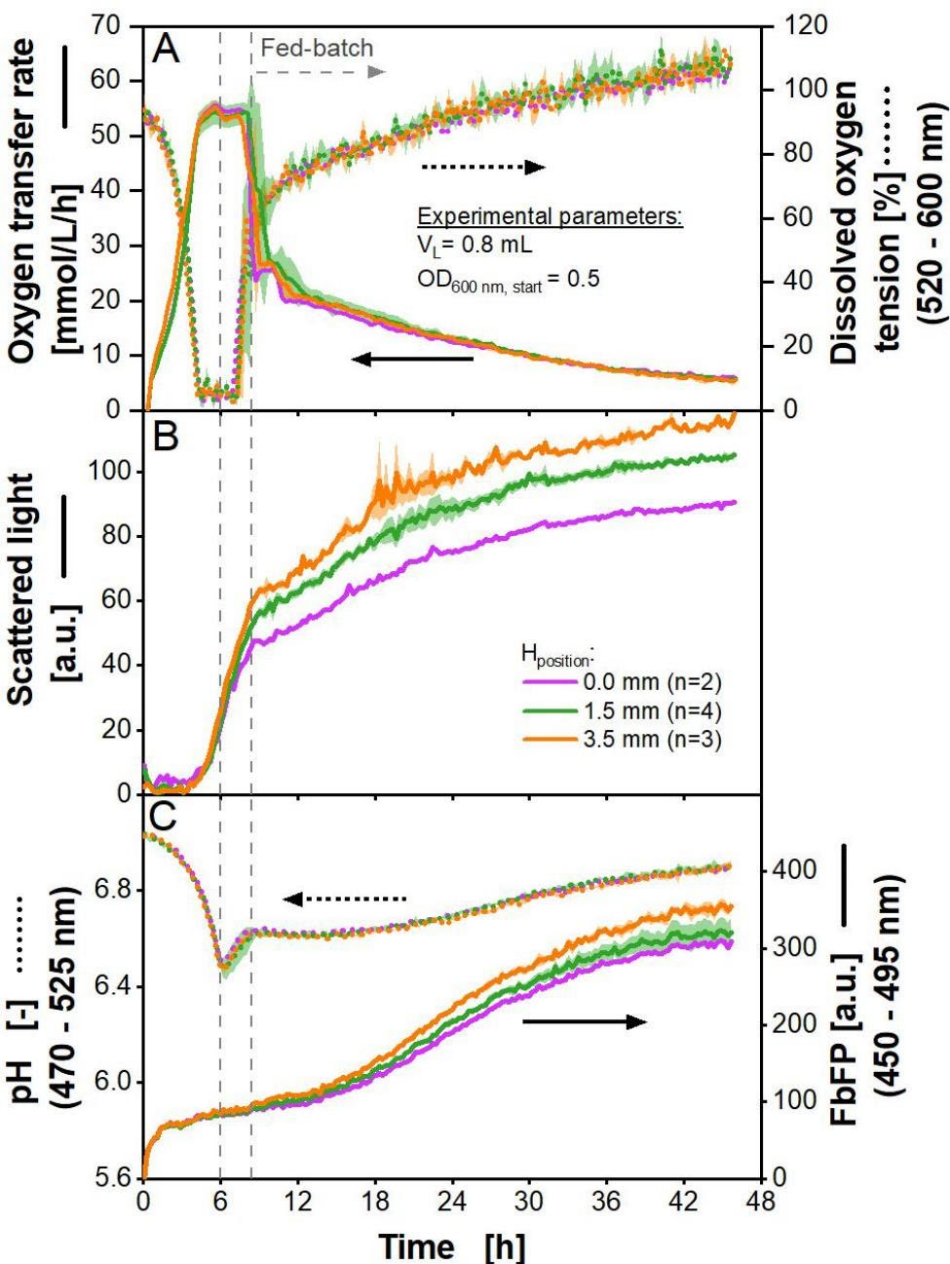
***E. coli *fed-batch cultivation using 48-well microtiter plates with black polymer rings containing glucose crystals.** Cultivation was performed in n replicates (given in Fig. 7B) with *E. coli* in Wilms-MOPS-medium using two microtiter plates, one with DOT and pH optodes (MTP-R48-B and MTP-R48-BOH). One plate was used for measurement in the BioLector, the other one for measurement in the µRAMOS-device. The first vertical grey dashed line marks the end of the batch phase. The second vertical grey line marks the beginning of the fed-batch phase with glucose release by the feed rings. Polymer rings (both plates): polymer rings with black dye and glucose crystals, d_inner_ = 8 mm, Z_height_ = 3 mm, H_position_ = 0 mm, 1.5 and 3.5 mm. Experimental parameters: initial pH = 7.5, V_L_ = 0.8 mL, T = 37 °C, n = 1000 rpm, d_0_ = 3 mm, OD_600 nm, start_ = 0.5. Commercial BioLector settings: Gain 15, λ_ex,em_ = 620 nm for scattered light, λ_ex_ = 520 nm and λ_em_ = 600 nm for DOT, λ_ex_ = 470 nm and λ_em_ = 525 nm for pH, λ_ex_ = 450 nm and λ_em_ = 495 nm for FbFP fluorescence, glass fibre in standard position (offset = 0 mm, Fig. S3A).

As a next step, biological fed-batch cultivations with *E. coli* with black glucose-releasing polymer rings were conducted. Product formation of EcFbFP was not actively induced. The OTR was measured using an in-house built µRAMOS device. The DOT, scattered light, pH and fluorescence were obtained using a commercial BioLector. The results are shown in Fig. 7.

The cultivation can be divided into three phases. The end of the first phase, the batch phase, is marked by the first vertical dashed grey line in the diagram. The batch phase is explained by the high initial glucose release, which is inherent for polymer-based materials. Additionally, at the beginning the initial biomass may be too low to immediately consume the released glucose [37, 38]. This batch phase is characterised by unlimited growth, visible in the exponential rise in the OTR (Fig. 7A) and mirrored in the exponentially decreasing DOT. The OTR and the DOT then reach a plateau, indicating an oxygen limitation [48]. The plateau of the DOT is around 5% and not as expected during an oxygen limitation at unmeasurable low values close to the K_m_-values for oxygen. This discrepancy may be due to deviations during calibration, as the plate manufacturer specifies a 5% accuracy range. The accuracy range would also explain that at the end of cultivation, the DOT slightly rises above 100% [68]. In Fig. 7B, the unlimited growth is also visible in the exponentially increasing scattered light signal. This leads to an increase in product formation, based on the fluorescence signal (Fig. 7C). Product formation was not actively induced, but the strain is known for the leaky expression of EcFbFP [6, 37, 69]. Product formation declines, when metabolic activity is limited by the oxygen supply. Elevated glucose concentration leads to the formation of overflow metabolites, such as acetate and lactate. This formation results in a sharp pH decrease, as seen in Fig. 7C [70, 71]. The initial pH is given with 7.5, but the measured data shows an initial pH value of 7.1. This might be partially explained with the accuracy range given by the manufacturer, which is ± 0.1 for pH values below 7.1 (no accuracy range is given for higher values) [68]. Apart from this, it is known that the pK_a_ value of MOPS buffer has a relatively high temperature dependency [72, 73]. The initial pH is set and measured at room temperature of 20 °C, while cultivation is conducted at 37 °C, leading to a lower measured pH.

The second phase, from 6 to 8.5 h, is dominated by the consumption of previously formed overflow metabolites after glucose is depleted, leading to an increase in pH (Fig. 7B). This takes place under oxygen limited conditions, as OTR and DOT are still at a plateau (Fig. 7A). Growth and product formation still increase, albeit at a lower rate than during the first phase due to less favourable carbon sources and oxygen limitation (Fig. 7B and C) [70].

The third phase is the fed-batch phase, characterised by the instantaneous consumption of glucose released by the polymer matrix, after the overflow metabolites have been consumed in phase two. Since glucose is only released in limited quantities through the matrix, the OTR decreases slowly, mirrored by a slowly increasing DOT. Another result of this limitation is a decline in the growth rate, detected by the declined increase in the scattered light signal (Fig. 7B). This may indicate the ongoing depletion of glucose in the polymer matrix. The fluorescence signal increases again, as more product is formed during fed-batch. Finally, as the metabolic activity declines at the end of the cultivation, the fluorescence reaches a plateau and protein is no longer produced (Fig. 7C).

All cultivations undergo these distinct phases, which are typical for fed-batch cultivations and have been previously described [35, 38, 74]. All measured values of the three investigated ring configurations align satisfactorily, except for the absolute scattered light value and the absolute fluorescence signal in the fed-batch phase. As breathing activity and pH align satisfactorily, it can be assumed that the cultures also run comparably in growth and product formation.

Fed-batch experiments with a higher initial optical density of 1.0 (Fig. S7) and a higher filling volume of 1.2 mL (Fig. S8) were conducted. The results are in accordance with the presented experiment above. An exception is the lower maximum oxygen transfer capacity, due to the higher filling volume in Figure S8A and D. Furthermore, scattered light and fluorescence reach lower values at the end of cultivation with a higher filling volume, indicating less biomass growth and product formation (Fig. S8B, C, E, F). In Figure S7B and C, as well as Figure S8B, C, E, and F, deviations for the scattered light signal and the fluorescence signal between the investigated ring configurations and filling volumes in the fed-batch phase are visible. As mentioned above, H_position_ and scattered light seem to correlate due to a measuring artefact, which could be confirmed with these experiments.

All investigated configurations are suitable to perform fed-batch cultivations. Due to the first initial glucose release of the polymer matrices, which is higher than the glucose consumption by the microorganisms, all fermentation experiments start with a batch phase. Such a batch phase is also common in the cultivation of *E. coli* in fermenters [75–77]. This ensures sufficient initial biomass, to produce the target product in a subsequent fed-batch phase. The duration of the batch-phase can be adjusted by choosing the initial optical density accordingly. Two different initial optical densities were investigated in this study. Raising the optical density from 0.5 to 1.0 allowed for earlier entry into fed-batch phase by approximately 2 h (Fig. 7A and S7A), which is in accordance with Habicher et al. (2019) [38]. Even higher initial optical densities should be investigated, to check the potential of the adjustability of the time point of entering the fed-batch phase. An earlier entry into fed-batch mode would potentially avoid oxygen limitation and its unfavourable characteristics, such as a decline in biomass growth and product formation as well as unwanted metabolic side effects. Future work on the feed rings may include alterations of the polymer matrix. The matrix composition influences the initial glucose burst, as well as the release rate [28].

### Applying polymer rings for biological fed-batch cultivations with the eukaryotic model organism *H. polymorpha*

**Fig. 8:**
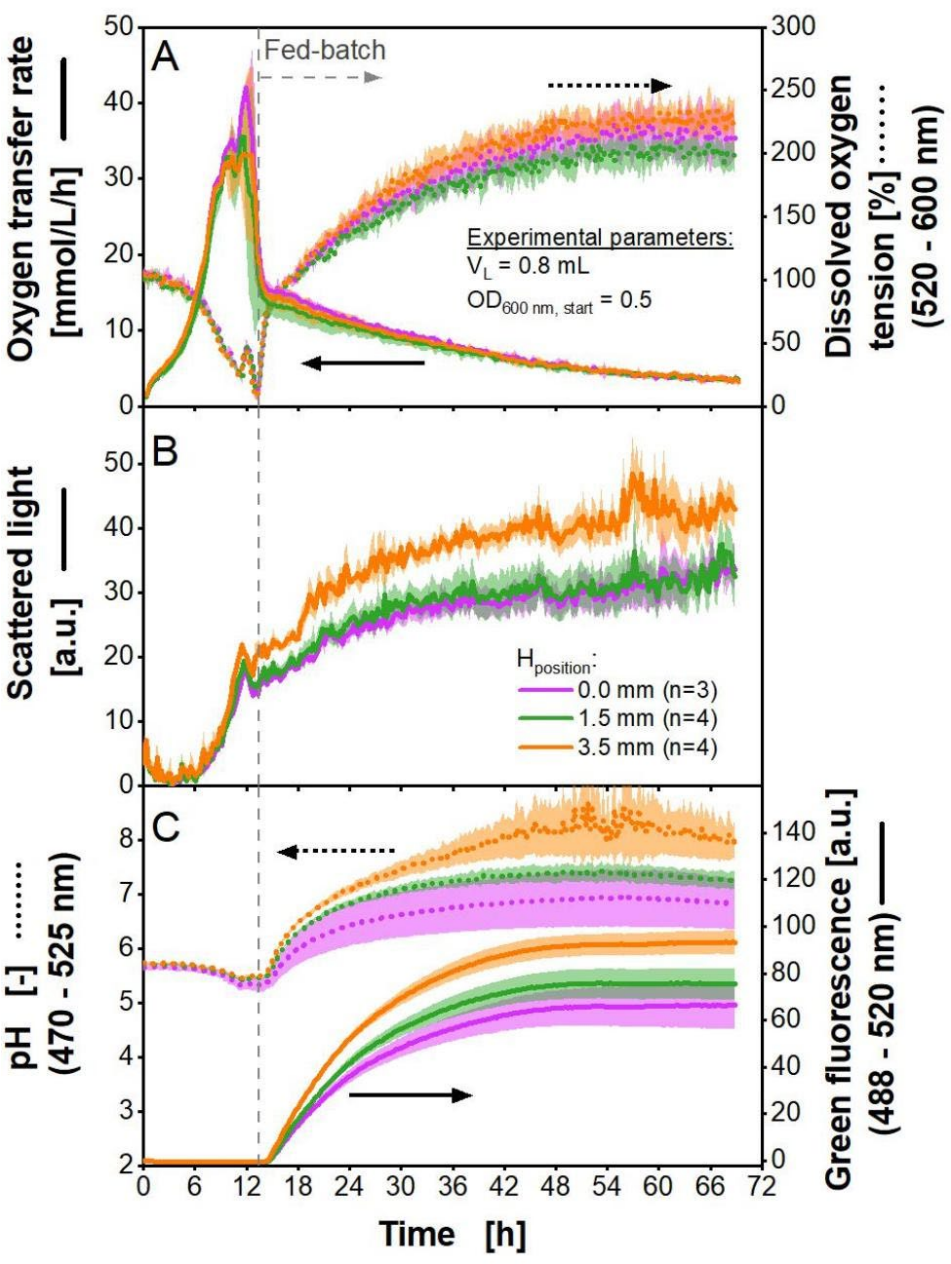
*** H. polymorpha *fed-batch cultivation using 48-well microtiter plates with black polymer rings containing glucose crystals.** Cultivation was performed in n replicates (given in Fig. 8B) with *H. polymorpha* in Syn6-MES medium using two microtiter plates, one with DOT and pH optodes (MTP-R48-B and MTP-R48-BOH). One plate was used for measurement in the commercial BioLector, the other one for measurement in the µRAMOS-device. The vertical grey dotted line marks the beginning of the fed-batch phase. Polymer rings (both plates): polymer rings with black dye and glucose crystals, d_inner_ = 8 mm, Z_height_ = 3 mm, H_position_ = 0 mm, 1.5 and 3.5 mm. Experimental parameters: initial pH = 6.0, V_L_ = 0.8 mL, T = 30 °C, n = 1000 rpm, d_0_ = 3 mm, OD_600 nm, start_ = 0.5. Commercial BioLector settings: Gain 15, λ_ex,em_ = 620 nm for scattered light, λ_ex_ = 520 nm and λ_em_ = 600 nm for dissolved oxygen tension (DOT), λ_ex_ = 470 nm and λ_em_ = 525 nm for pH, λ_ex_ = 488 nm and λ_em_ = 520 nm for green fluorescence, glass fibre in standard position (at for e.g., well A1 at X = 14 mm and Y = 75 mm, offset = 0 mm, Figure S3A).

To prove the working principle and suitability of the fed-batch polymer rings, fed-batch cultivations with a second model organism, the yeast *H. polymorpha*, producing a green fluorescent protein (GFP), were conducted. The results are shown in Fig. 8.

The cultivation can be divided into a batch phase and a fed-batch phase, marked in the diagram with a vertical dashed grey line. In the batch phase, the biomass concentration is yet insufficient to consume immediately all the released glucose by the matrix, as described earlier. During this phase, the OTR rises exponentially until a double peak is observed (Fig. 8A). The first peak is formed by the consumption of glucose. The second peak indicates the consumption of overflow metabolites such as ethanol [36]. The evolution of the OTR is mirrored by the DOT. Scattered light increases in the batch phase, indicating exponential biomass growth (Fig. 8B). The pH decreases due to glucose consumption (Fig. 8C) [70]. As long as the initial excess of glucose is consumed, GFP production is repressed and no increase in green fluorescence signal is observed [37].

In the fed-batch phase, the OTR decreases slowly, mirrored by a slowly increasing DOT (Fig. 8A). Biomass formation declines, as the scattered light signal increases at a lower rate than in the exponential growth phase (Fig. 8B). The production of GFP starts to increase in the fed-batch phase, accompanied by an increase in the pH (Fig. 8C). The measurements of both parameters (pH and GFP) can interfere with each other, due to similar emission wavelengths of the pH optode and GFP. Hence, the increase in the pH signal might be promoted by the increasing GFP signal. This phenomenon has been described before [41]. After approximately 42 h, the fluorescence signal reaches a plateau, and no further product formation is visible. At this time point, the glucose in the polymer matrices is probably depleted. This is also indicated by the slow decreasing OTR. The remaining glucose release is predominately consumed for cell maintenance.

The DOT increase to values up to over 200% towards the end of cultivation might be explained by interferences with the wavelength of fluorescence measurement, as the wavelengths overlap at around 520 nm (Fig. 8A and C). With ongoing cultivation time, an increase in deviation of the pH and fluorescence signal is notable. Again, this might be due to the interferences of the similar emission wavelengths, as discussed above.

All three investigated polymer ring configurations undergo the above-mentioned characteristic changes over time. After an initial batch phase of approx. 12 h, all cultivations show the previously described distinct fed-batch phase [36]. The trends in the scattered light and fluorescence signal of wells with different H_positions_ are similar to the one observed for fed-batch *E. coli* cultivations (Fig. 7B and C, S7B and C, S8B, C, E, F). Therefore, the differences may be caused by measurement interferences rather than biological phenomena, though, they could not be observed in abiotic experiments (Fig. 5). Interestingly, the DOT, which is also measured via the bottom of the plate, is not significantly affected. Until now, the cause of this observation could not be identified.

A higher initial density of 1.0 was also investigated and the results can be seen in Figure S9. The batch phase could be reduced by 4 h, compared to cultivations with an initial optical density of 0.5. The results for fed-batch cultivations with a filling volume of 1.2 mL per well are in accordance with the above presented findings (Fig. S10).

### Determination of the glucose release

**Fig. 9:**
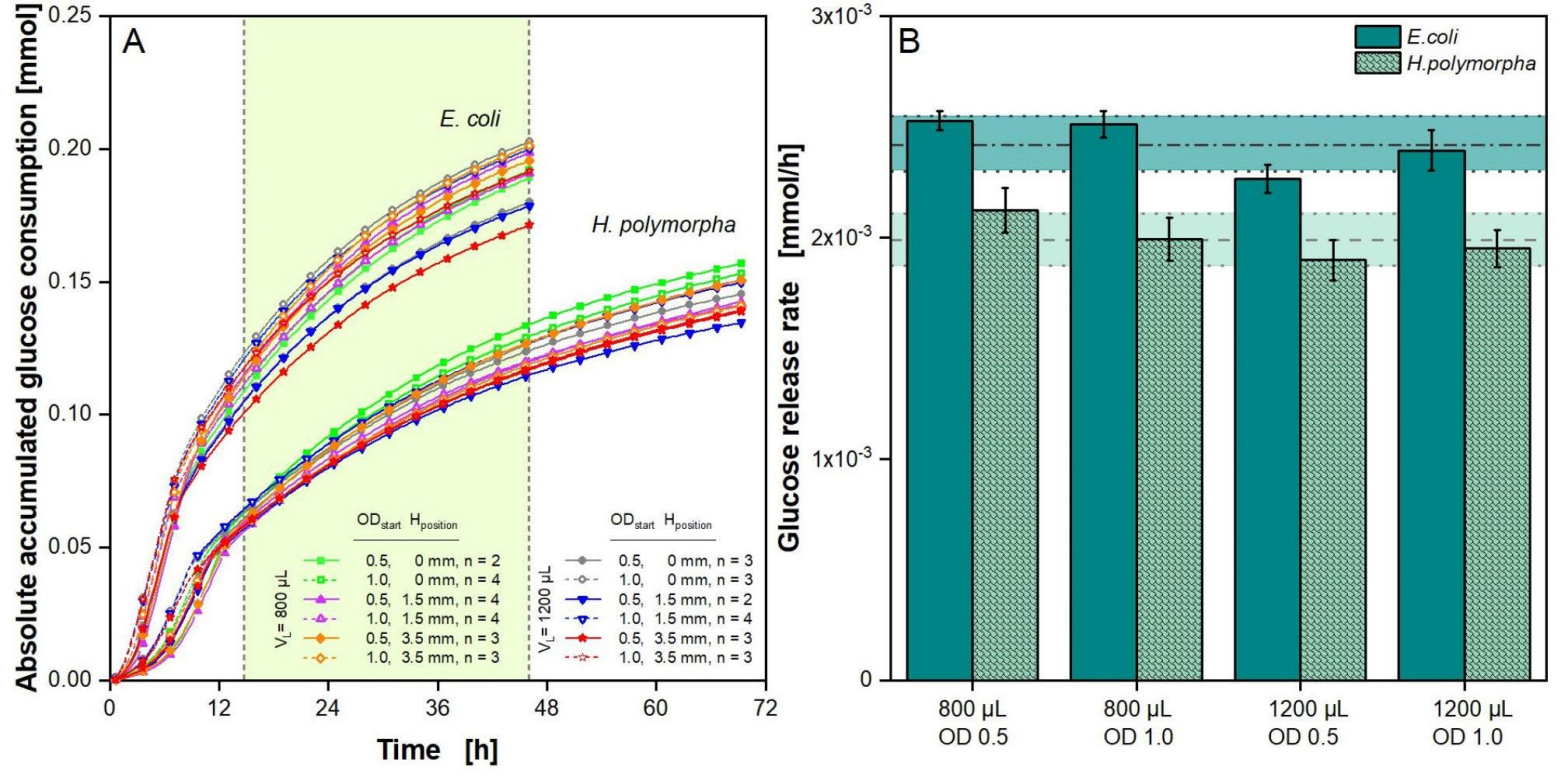
**Absolute accumulated glucose consumption and glucose release rates based on oxygen transfer rates.** (A) Absolute accumulated glucose consumption for *E. coli* and *H. polymorpha* fed-batch cultivations. Glucose consumption was calculated with Eqs. (3)-(5) based on the oxygen consumption (Figure S11). Only every fifth point is shown for clarity. (B) Based on the absolute accumulated glucose consumption (Fig. 9A), the glucose release rates were determined for the fed-batch phase for the time frame, that is marked in Fig. 9A as the light green area between the two vertical, dotted lines. The slope of the linear fit within this time frame equals the glucose release rate. Mean values were calculated for cultivations with one polymer ring (Z_height_ = 3 mm) in three different positions (H_position_ = 0 mm, 1.5 and 3.5 mm) with the same filling volume (V_L_ = 0.8 mL and 1.2 mL) and initial optical density (OD_600 nm, start_ = 0.5 and 1.0). The horizontal grey dashed and dotted line marks the mean value of results from *E. coli* cultivations, while the horizontal grey dashed line marks the mean value from results from *H. polymorpha* cultivations. The coloured area depicts the respective standard deviation. Polymer rings: polymer rings with black dye and glucose crystals, d_inner_ = 8 mm, Z_height_ = 3 mm, H_position_ = 0 mm, 1.5 and 3.5 mm. Experimental parameters: initial pH = 7.5 (*E. coli)* and 6.0 (*H. polymorpha*), V_L_ = 0.8 mL and 1.2 mL, T = 37 °C (*E. coli)* and 30 °C *(H. polymorpha)*, n = 1000 rpm, d_0_ = 3 mm, OD_600 nm, start_ = 0.5 and 1.0

The online data obtained in the fed-batch experiments presented in the two previous sections was evaluated, to calculate the absolute glucose release rate as described in the Materials and Methods section. Based on accumulated oxygen transfer (Fig. S11), the absolute glucose consumption for fed-batch cultivations with *E. coli* and *H. polymorpha* was calculated and then plotted against time, shown in Fig. 9A. Cultivation time varies depending on the organism, which have varying metabolism and growth rates. For the fed-batch phase, a decrease in slope over time is observable, which may hint towards a depletion of glucose in the polymer matrix. Both model organisms show slight variations of up to 0.03 mol for the absolute glucose consumption. This variation might originate from biological variation, as no trend regarding filling volume, polymer ring configuration or initial optical density can be observed. Furthermore, the glucose consumption for *H. polymorpha* is approximately 0.05 mmol lower than for *E. coli* cultivations. This is independent of any variations in filling volume, ring configuration or initial optical density.

For a more accurate comparison, the glucose release rate was calculated from Fig. 9A from the highlighted light green area, which marks the common time phase both organisms spend in the fed-batch phase. During the fed-batch phase, glucose is limiting, as glucose released by the polymer matrix is immediately consumed by the microorganisms. Hence, the glucose consumption equals the glucose release by the polymer matrix. A linear fit was used to determine the slope of the curves, which equals the glucose release rate. Glucose release rates for cultivations with both model organisms are shown in Fig. 9B. Mean values were calculated from all polymer ring configurations with the same filling volume and initial optical density. Additionally, the mean value for all conditions, filling volumes and initial optical densities was calculated for each organism. It is marked for *E. coli* by the dark grey dotted and dashed line and for *H. polymorpha* by the light grey dashed line with the deviation marked by the coloured area. Corresponding to the findings of Fig. 9A, a difference in glucose release rate is observable between both organisms. *E. coli* cultivations show a higher mean glucose release rate of 0.0024 mmol/h, compared to *H. polymorpha* cultivations, showing a mean glucose release rate of 0.0020 mmol/h. Hence, the ratio of glucose release in *E. coli* and *H. polymorpha* cultivations is 1.22.

Similar glucose release rates for all cultivations were expected, as the same polymer-matrix was applied. The polymer matrix has been characterized before by Keil et al. (2019) and showed a dependency on cultivations conditions [37]. They fitted the glucose release rates in microtiter plates with Wilms-MOPS medium as function of osmotic concentration, pH, temperature and initial ammonium concentration. They also showed, that Syn6-MES medium behaved comparable to Wilms-MOPS medium regarding glucose release and its dependency on temperature. Therefore, it was assumed that the application of the fits would be appropriate to calculate the glucose release rates for the *E. coli* and *H. polymorpha* cultivations in this work and compare them to one another.

The glucose release rates for the four parameters osmotic concentration, pH, temperature and initial ammonium concentration were calculated based on the fits of Keil et al. (2019) with the parameters used within this work. The temperature was constant throughout cultivation (*E. coli*: 310,15 K, *H. polymorpha*: 303,15 K) (It must be noted that there is a printing error in the publication by Keil et al. (2019), which was confirmed by the authors through personal communication. The fit parameter b for the correlation between temperature and glucose release is 1.41·10^5^ and not 1.41·10^8^ [37]). Osmotic concentration as well as initial ammonium concentration were determined from the composition of fresh medium (*E. coli*: 582 mOsmol/kg and 14.4 mmol/L initial ammonium, *H. polymorpha*: 512 mOsmol/kg and 15.8 mmol/L initial ammonium). For pH, the pH value during the common fed-batch phase (time span used for calculation of glucose release rates, Fig. 9A) was used (*E. coli*: 6.7 on average, *H. polymorpha*: 10% of the time ≤ 6.5, 90% of the time > 6.5). For both organisms, the product of the four glucose release rates was calculated and the ratio determined. The ratio of the product of the glucose release rates of *E. coli* and *H. polymorpha* is 1.16. This is similar to the ratio of 1.22 calculated from the cultivation data. Hence, it can be stated that the differences in glucose consumption and glucose release rates are mainly due to the release kinetics of the polymer matrix, influenced by the varying cultivation conditions.

Characterizations of the matrix used in this work have already been published. Habicher et al. (2019) investigated polymer rings at the bottom of the well and determined glucose release rates by measuring the glucose content over time in wells conducting abiotic experiments in Wilms-MOPS-medium [38]. They obtained a glucose release rate of 0.37 mg/h, similar to the one obtained in this study for *H. polymorpha* (0.36 mg/h ± 0.02 mg/h), but slightly lower than for the results for *E. coli* (0.44 mg/h ± 0.02 mg/h). In the publication by Keil et al. (2020), the glucose release rate over time for their standard matrix has been abiotically measured [28]. Their standard matrix is identical to the matrix used in this study, but fully covered the bottom of the well. They determined a glucose release rate of 0.24 mg/h. The values obtained within this work of 0.36 and 0.44 mg/h are approximately 1.5 to 1.8 times higher. The difference may be explained via the different matrix forms, as that influences the contact area of medium and matrix and, with that, the glucose release rate. Furthermore, the experimental setup and the chosen parameters for all determination methods are not identical (for example the temperature, 30 °C vs. 37 °C) and may influence the results. A major difference is that both characterization methods were done abiotically, while in this work the glucose release rate was calculated based on biological data. Nonetheless, all glucose release rates lay within a comparable range. Thus, the polymer rings in the investigated configurations can successfully be used for fed-batch cultivations.

## Conclusion

Within this work, it could be shown that fed-batch cultivations with polymer feed rings incorporated into commercial microtiter plates with online measurement in the µRAMOS as well as in a commercial BioLector are possible. Various polymer ring configurations (inner ring diameters of 7 and 8 mm as well as ring heights (Z_height_) of 3, 6 and 9 mm at heights in the well (H_position_) of 0, 1.5, 3.5 and 5.5 mm) were investigated regarding oxygen availability, mixing time and interference with scattered light measurements. Favourable configurations, which allow comparable measurements to wells without polymer rings, are in particular the following for both inner diameters: a Z_height_ of 3 or 6 mm at H_positions_ of 0, 1.5 and 3.5 mm. H_position_ = 5.5 mm only works for a Z_height_ of 3 mm. To keep the manufacturing process simple, H_position_ = 0 mm seems to be the most beneficial configuration. By changing the ring colour from white to black, the reflected light in the well could be reduced to a level equal to wells without polymer rings. This allows for measurements in a commercial BioLector without adjustment of the relative measurement position of the optical fibres.

The applicability of the polymer rings was demonstrated in fed-batch cultivations with two model organisms, *E. coli* and *H. polymorpha*, applying three different black polymer ring configurations (Z_height_ = 3 mm, H_position_ = 0, 1.5, and 3.5 mm), as well as two initial optical densities (0.5 and 1.0) and filling volumes (800 µL and 1200 µL). With all given parameter variations, comparable, parallel measurements of dissolved oxygen tension, scattered light, pH, fluorescence and oxygen transfer rate were possible. Due to a high initial glucose burst from the polymer matrix, a batch phase is visible at the beginning of the cultivation. This might come as an advantage, as enough biomass formation is ensured, before product formation occurs. By using a higher initial optical density, the initial batch phase can be shortened.

The calculated glucose release rates for each configuration used for fed-batch cultivations with two model organisms are comparable with each other and with previously published data of the same matrix. Observed differences for the glucose release between the two model organisms could be shown to originate from the matrix glucose release, which varies as function of temperature, pH and medium composition. The application of different polymer matrix materials should be part of future investigations for a broader range of release rates.

As fed-batch cultivations are state of the art for industrial processes, fed-batch cultivations in microtiter plates allow for effective strain and medium screening with online measurement. Labour-intensive end-point measurement or even time course sampling throughout cultivation can be avoided. Based on the acquired data during this work, the usage of polymer rings for fed-batch cultivations in microtiter plates could be optimised to allow for adjustment-free measurement in a commercial BioLector. During cultivation, signals such as DOT, scattered light, pH and fluorescence are available. Paired with a µRAMOS, it is possible to additionally measure the OTR. Thus, the presented results introduce an easy, adjustment-free fed-batch system in microtiter plates with online measurement.

## Electronic supplementary material

Below is the link to the electronic supplementary material.

Supplementary Material 1

## Data Availability

The datasets used and analysed during the current study are available from the corresponding author on reasonable request.

## Abbreviations

AOT: Accumulated oxygen transfer
d_inner_: Inner polymer ring diameter
d_outer_: Outer polymer ring diameter
FbFP: Flavin-based fluorescent protein
IPTG: Isopropyl β-d-1-thiogalactopyranoside
GFP: Green fluorescent protein
H_position_: Polymer ring position within the well
HTPS: High throughput system
MTP: Microtiter plate
DOT: Dissolved oxygen tension
OD_600nm_: Optical density at 600 nm
OTR: Oxygen transfer rate
OTR_max_: Maximum oxygen transfer capacity
OTR_raw_: Raw oxygen transfer rate calculated by the µRAMOS software
OTR_real_: Volume-corrected oxygen transfer rate
V_L_: Liquid filling volume in the well
V_polymer ring_: Polymer ring volume
V_well_: Well volume
Z_height_: Polymer ring height
