# Supplementary material for: Black glucose-releasing silicon elastomer rings for fed-batch operation allow measurement of the oxygen transfer rate from the top and optical signals from the bottom for each well of a microtiter plate
Source: BMC Biotechnol. 2023 Mar 2;23:5. doi: 10.1186/s12896-023-00775-9 (PMC9983259; doi:10.1186/s12896-023-00775-9)
Supplement: Supplementary file 1 — Supplementary Material 1 [file 12896_2023_775_MOESM1_ESM.docx]

**Supplementary Data**

**Manuscript:**

**Black glucose-releasing silicon elastomer rings for fed-batch operation allow measurement of the oxygen transfer rate from the top and optical signals from the bottom for each well of a microtiter plate**

**Sarah Sparviero^a^, Laura Barth^a^, Timm Keil^a^, Carl Dinter^a^, Christoph Berg^a^, Clemens Lattermann^b^ and Jochen Büchs^a,*^**

^a^ Aachener Verfahrenstechnik – Biochemical Engineering, RWTH Aachen University, Forckenbeckstr. 51, 52074 Aachen, Germany

^b^ Kuhner Shaker GmbH, Kaiserstraße 100, 52134 Herzogenrath, Germany

^*^ Corresponding author:

Prof. Dr.-Ing. Jochen Büchs, RWTH Aachen University, Aachener Verfahrenstechnik – Chair of Biochemical Engineering, Bldg. NGP^2^, Forckenbeckstr. 51, 52074 Aachen, Germany; Phone: +49 (0) 241 – 80-24633; Fax: +49 (0) 241 80 22635; E-mail: jochen.buechs@avt.rwth-aachen.de





**Figure S1:** **Biological determination principle of the maximum oxygen transfer capacity (OTR_max_) with polymer rings.** Cultivations were conducted in 48 round well microtiter plates in triplicates (n = 3) with *E.* *coli* in Wilms-MOPS medium with 20 g/L glucose. The oxygen transfer rate (OTR) was determined using the µRAMOS - device. Cultivation conditions: initial pH = 7.5, OD_600_ _nm,_ _start_ = 0.5, V_L_ = 0.8 mL, T = 37 °C, n = 1000 rpm, d_0_ = 3 mm. Transparent polymer rings without glucose crystals (Fig. 2A): d_inner_ = 8 mm. Two exemplary configurations are shown: H_position_ = 1.5 mm and 5.5 mm; Z_height_ = 6 mm and 9 mm.





**Figure S2:** **Exemplary presentation of the determination of the mixing time by the MATLAB^®^-tool.** The recorded video files of the colour change in shaking MTPs are split into the respective red, green and blue channels. For further processing, only the red channel is used, as it shows the largest change during the colour change. For each frame, the 90^th^ and 10^th^ percentile of the red channel were calculated and fitted to a sigmoidal curve. To minimize the data that needs to be processed, only every 20^th^ frame was used for calculation. The start of the colour change equals the beginning of the sigmoidal slope of the 90^th^ percentile, while the end of the colour change is depicted by the end of the slope of the fit of the 10^th^ percentile. The mixing time was then calculated from the number of frames between the start and the end of the colour change and the frame rate of the GoPro camera, which is 240 frames per second.

**
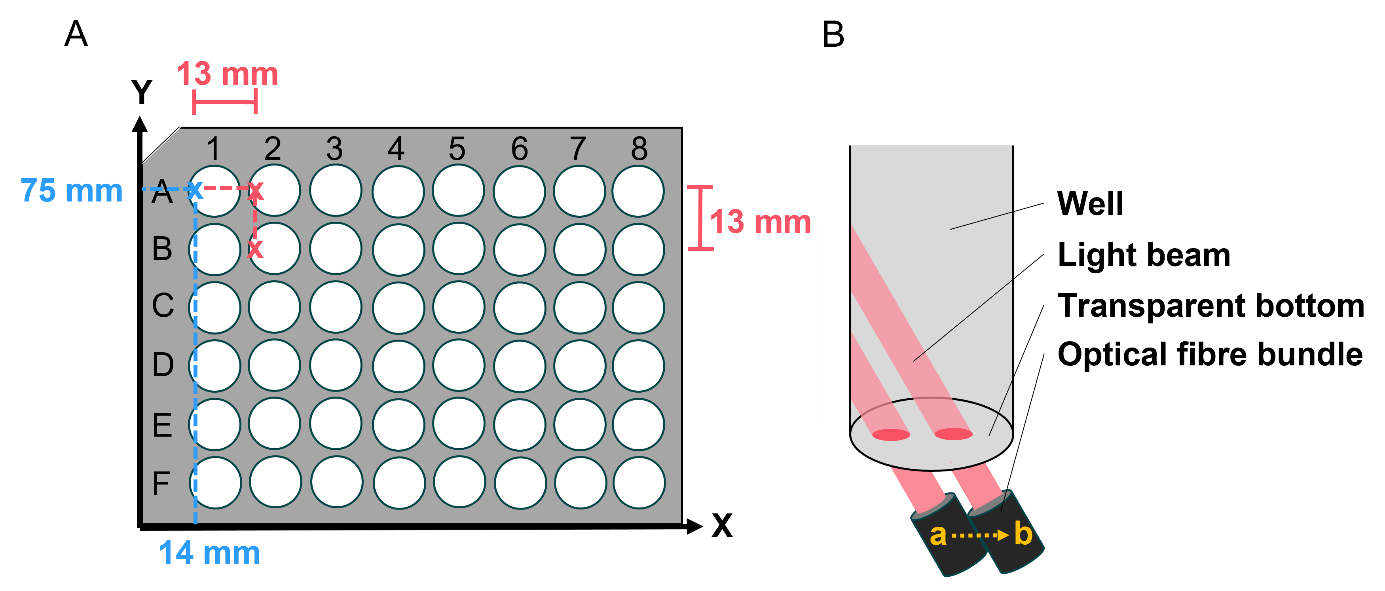
**

**Figure S3:** **Schematic view of the coordinate system of the X-Y-positioning device and a well’s side view.** (A) For the basis of the coordinate system, the commercial BioLector software uses the frame of a 48-well microtiter plate (MTP-R48-series). The bottom left corner of the plate represents the origin of this coordinate system. The default measurement position for well A1 is X = 14 mm and Y = 75 mm (shown in blue colour). From this position, the X-Y-positioning device moves 13 mm in either X- or Y- direction to measure at the same relative position within each well (shown for well A2 and B2 as example in red colour). (B) Schematic side view of a well, showing the light beam of the optical fibre bundle entering the well through the transparent bottom. The orange dotted arrow schematically shows the change in measuring position of the glass fibre from default position a to an offset measurement position b.





**Figure S4: Influence of polymer rings on the maximum oxygen transfer capacity (OTR_max_) and the mixing time.** 48 round well microtiter plate, polymer rings: transparent polymer rings without glucose crystals (Fig.  2A), d_inner_= 7mm, H_position_ = 0.0 mm, 1.5 mm, 3.5 mm and 5.5 mm, Z_height_ = 3 mm, 6 mm and 9 mm. As a control, wells without any polymer rings were evaluated, highlighted in the diagram with a purple dashed line ( - - - ). (A) Influence of polymer ring configurations on the OTR_max_. Values were determined for cultivations with *E.* *coli* in Wilms-MOPS medium with 20 g/L glucose conducted in triplicates. Error bars depict the standard deviation, calculated for each set of triplicates. The oxygen transfer rate (OTR) was determined using the µRAMOS. Cultivation conditions: initial pH = 7.5, OD_600_ _nm,_ _start_ = 0.5, V_L_ = 0.8 mL, T = 37 °C, n = 1000 rpm, d_0_ = 3 mm. (B) Influence of polymer ring configurations on the mixing time. The mixing time was determined in triplicates with a colour change method, illustrated in Figure 3. Error bars represent the standard deviation, calculated for each set of triplicates. Experimental conditions: V_L_ = 0.8 mL, T = 37 °C, n = 1000 rpm, d_0_ = 3 mm.

**

 Figure S5: Influence of H_position_ of differently coloured polymer rings on measurement of scattered light in a commercial BioLector device.** Measurements were performed in 48 round well microtiter plates . Polymer rings: (A) white polymer rings with glucose crystals (Fig. 2B) and (B) rings without glucose crystals sprayed with black lacquer (Fig. 2C), d_inner_= 7 mm, Z_height_ = 3 mm, H_position_ = 0.0 mm, 1.5 mm, 3.5 mm and 5.5 mm. As a control, wells without polymer rings were evaluated. Experimental parameters: V_L_ = 0 mL, T = 37 °C, n = 1000 rpm, d_0_ = 3 mm. Commercial BioLector settings: Gain 15, λ_ex,_ _em_ = 620 nm, three replicates measured three times, position of the X-Y-positioning device (glass fibre) was varied on the X-axis (Fig.  S3) with a positive offset of 0 to 6.5 mm relative to the standard position.





**Figure S6: Influence of the H_position_ of a black polymer ring on measurement of scattered light.** Cultivation was performed with *E.* *coli* in Wilms-MOPS-medium with 20 g/L glucose using a 48-well microtiter plate (MTP-R48-B) in triplicates (n = 3). Polymer rings: rings without glucose crystals sprayed with black lacquer (Fig. 2C), d_inner_ = 7 mm, Z_height_ = 3 mm, H_position_ = 0.0 mm, 1.5 mm, 3.5 mm and 5.5 mm. As a control, wells without polymer rings were evaluated. Experimental parameters: initial pH = 7.5, V_L_ = 0.8 mL, T = 37 °C, n = 1000 rpm, d_0_ = 3 mm, OD_600 nm, start_ = 0.5. Commercial BioLector settings: Gain 15, λ_ex,_ _em_ = 620 nm, glass fibre in standard position (offset = 0 mm, Fig. S3).





**Figure S7: *E.* *coli* fed-batch cultivation with black polymer rings and an initial optical density of 1.0.** Cultivation was performed in n replicates with *E.* *coli* in Wilms-MOPS-medium using two 48-well microtiter plates, one with DOT and pH optodes (MTP-R48-B and MTP-R48-BOH). One plate was used for measurement in a commercial BioLector, the other one for measurement in the µRAMOS-device. The first vertical grey dashed line marks the end of the batch phase with growth on excessive glucose. The second vertical grey line marks the beginning of the fed-batch phase with glucose release by the feed rings. Polymer rings (both plates): polymer rings with black dye with glucose crystals, d_inner_ = 8 mm, Z_height_ = 3 mm, H_position_ = 0 mm, 1.5 mm and 3.5 mm. Experimental parameters: initial pH = 7.5, V_L_ = 0.8 mL, T = 37 °C, n = 1000 rpm, d_0_ = 3 mm, OD_600_ _nm,_ _start_ = 1.0. Commercial BioLector settings: Gain 15, λ_ex_, _em_ = 620 nm for scattered light, λ_ex_ = 520 nm and λ_em_ = 600 nm for dissolved oxygen tension (DOT), λ_ex_ = 470 nm and λ_em_ = 525 nm for pH, λ_ex_ = 450 nm and λ_em_ = 495 nm for FbFP fluorescence, glass fibre in standard position (offset = 0 mm, Fig. S3A).





**Figure S8: *E.* *coli* fed-batch cultivations with black polymer rings (filling volume per well = 1.2 mL).** (A-C) OD_600_ _nm,_ _start_ = 0.5, (D-F) OD_600_ _nm,_ _start_ = 1.0. Cultivation was performed in n replicates with *E.* *coli* in Wilms-MOPS-medium using two 48-well microtiter plates, one with DOT and pH optodes (MTP-R48-B and MTP-R48-BOH). One plate was used for measurement in a commercial BioLector, the other one for measurement in the µRAMOS-device. The first vertical grey dashed line marks the end of the batch phase with growth on excessive glucose. The second vertical grey line marks the beginning of the fed-batch phase with glucose release by the feed rings. Polymer rings (both plates): polymer rings with black dye with glucose crystals, d_inner_ = 8 mm, Z_height_ = 3 mm, H_position_ = 0 mm, 1.5 mm and 3.5 mm. Experimental parameters: initial pH = 7.5, V_L_ = 1.2 mL, T = 37 °C, n = 1000 rpm, d_0_ = 3 mm, OD_600 nm, start_ = 0.5 and 1.0. Commercial BioLector settings: Gain 15, λ_ex,em_= 620 nm for scattered light, λ_ex_= 520 nm and λ_em_= 600 nm for dissolved oxygen tension (DOT), λ_ex_= 470 nm and λ_em_= 525 nm for pH, λ_ex_= 450 nm and λ_em_= 495 nm for FbFP fluorescence, glass fibre in standard position (offset = 0 mm, Fig. S3A).





**Figure S9: *H.*** ***polymorpha* fed-batch cultivation with black polymer rings and an initial optical density of 1.0.** Cultivation was performed in n replicates with *H.* *polymorpha* in Syn6-MES medium using two 48-well microtiter plates, one with DOT and pH optodes (MTP-R48-B and MTP-R48-BOH). One plate was used for measurement in a commercial BioLector, the other one for measurement in the µRAMOS. The vertical grey dashed line marks the beginning of the fed-batch phase, after consumption of the excessive glucose. Polymer rings (both plates): polymer rings with black dye with glucose crystals, d_inner_ = 8 mm, Z_height_ = 3 mm, H_position_ = 0 mm, 1.5 mm and 3.5 mm. Experimental parameters: initial pH = 6.0, V_L_ = 0.8 mL, T = 30 °C, n = 1000 rpm, d_0_ = 3 mm, OD_600_ _nm,_ _start_ = 1.0. Commercial BioLector settings: Gain 15, λ_ex,_ _em_ = 620 nm for scattered light, λ_ex_ = 520 nm and λ_em_ = 600 nm for dissolved oxygen tension (DOT), λ_ex_ = 470 nm and λ_em_ = 525 nm for pH, λ_ex_ = 488 nm and λ_em_ = 520 nm for green fluorescence, glass fibre in standard position (offset = 0 mm, Fig. S3A).





**Figure S10: *H.*** ***polymorpha* fed-batch cultivations with black polymer rings (filling volume per well = 1.2** **mL).** (A-C) OD_600_ _nm,_ _start_ = 0.5, (D-F) OD_600_ _nm,_ _start_ = 1.0. Cultivation was performed in n replicates with *H.* *polymorpha* in Syn6-MES medium using two 48-well microtiter plates, one with DOT and pH optodes (MTP-R48-B and MTP-R48-BOH). One plate was used for measurement in a commercial BioLector, the other one for measurement in the µRAMOS-device. The vertical grey dotted line marks the beginning of the fed-batch phase, after consumption of the excessive glucose. Polymer rings (both plates): polymer rings with black dye with glucose crystals, d_inner_= 8 mm, Z_height_ = 3 mm, H_position_ = 0 mm, 1.5 mm and 3.5 mm. Experimental parameters: initial pH = 6.0, V_L_ = 1.2 mL, T = 30 °C, n = 1000 rpm, d_0_ = 3 mm. Commercial BioLector settings: Gain 15, λ_ex,_ _em_ = 620 nm for scattered light, λ_ex_ = 520 nm and λ_em_ = 600 nm for dissolved oxygen tension (DOT), λ_ex_ = 470 nm and λ_em_ = 525 nm for pH, λ_ex_ = 488 nm and λ_em_ = 520 nm for green fluorescence, glass fibre in standard position (offset = 0 mm, Fig. S3A).

**

**

**Figure S11: Accumulated oxygen transfer (AOT) of *E.*** ***coli* and *H.*** ***polymorpha* fed-batch cultivations.** (A) Cultivation was performed in n replicates with *E.* *coli* in Wilms-MOPS-medium using the µRAMOS-device. (B) Cultivation was performed in n replicates with *H.* *polymorpha* in Syn6-MES medium using the µRAMOS-device. 48 round well microtiter plates, polymer rings with black dye with glucose crystals, d_inner_ = 8 mm, Z_height_ = 3 mm, H_position_ = 0 mm, 1.5 mm and 3.5 mm. Experimental parameters *E.* *coli* cultivations: initial pH = 7.5, V_L_ = 0.8 mL and 1.2 mL, T = 37 °C, n = 1000 rpm, d_0_ = 3 mm, OD_600_ _nm,_ _start_ = 0.5 and 1.0. Experimental parameters *H.* *polymorpha* cultivations: initial pH = 6.0, T = 30 °C, n = 1000 rpm, d_0_ = 3 mm, OD_600_ _nm,_ _start_ = 0.5 and 1.0. Standard deviation is only shown for cultivations with n ≥ 3. Only every tenth point is shown for clarity. The corresponding oxygen transfer rates, from which the accumulated oxygen transfer was calculated by integration, can be seen in Figure 7, 8 and S7 – S10.
